# Comparison of the ability of exosomes and ectosomes derived from adipose-derived stromal cells to promote cartilage regeneration in a rat osteochondral defect model

**DOI:** 10.1186/s13287-024-03632-4

**Published:** 2024-01-17

**Authors:** Tengjing Xu, Xinning Yu, Kaiwang Xu, Yunting Lin, Jiajie Wang, Zongyou Pan, Jinghua Fang, Siheng Wang, Zhuxing Zhou, Hongyun Song, Sunan Zhu, Xuesong Dai

**Affiliations:** 1 Department of Orthopedic Surgery, The Second Affiliated Hospital, Zhejiang University School of Medicine, Hangzhou City, Zhejiang Province People’s Republic of China; 2 Orthopedics Research Institute of Zhejiang University, Hangzhou City, Zhejiang Province People’s Republic of China; 3 Key Laboratory of Motor System Disease Research and Precision Therapy of Zhejiang Province, Hangzhou City, Zhejiang Province People’s Republic of China; 4 Clinical Research Center of Motor System Disease of Zhejiang Province, Hangzhou City, People’s Republic of China

**Keywords:** Cartilage regeneration, Mesenchymal stromal cells, Exosomes, Ectosomes, Macrophages

## Abstract

**Background:**

Extracellular vesicles (EVs) derived from mesenchymal stromal cells (MSCs) offer promising prospects for stimulating cartilage regeneration. The different formation mechanisms suggest that exosomes and ectosomes possess different biological functions. However, little attention has been paid to the differential effects of EV subsets on cartilage regeneration.

**Methods:**

Our study compared the effects of the two EVs isolated from adipose-derived MSCs (ASCs) on chondrocytes and bone marrow–derived MSCs (BMSCs) in vitro. Additionally, we loaded the two EVs into type I collagen hydrogels to optimize their application for the treatment of osteochondral defects in vivo.

**Results:**

In vitro experiments demonstrate that ASC-derived exosomes (ASC-Exos) significantly promoted the proliferation and migration of both cells more effectively than ASC-derived ectosomes (ASC-Ectos). Furthermore, ASC-Exos facilitated a stronger differentiation of BMSCs into chondrogenic cells than ASC-Ectos, but both inhibited chondrocyte apoptosis to a similar extent. In the osteochondral defect model of rats, ASC-Exos promoted cartilage regeneration in situ better than ASC-Ectos. At 8 weeks, the hydrogel containing exosomes group (Gel + Exo group) had higher macroscopic and histological scores, a higher value of trabecular bone volume fraction (BV/TV), a lower value of trabecular thickness (Tb.Sp), and a better remodeling of extracellular matrix than the hydrogel containing ectosomes group (Gel + Ecto group). At 4 and 8 weeks, the expression of CD206 and Arginase-1 in the Gel + Exo group was significantly higher than that in the Gel + Ecto group.

**Conclusion:**

Our findings indicate that administering ASC-Exos may be a more effective EV strategy for cartilage regeneration than the administration of ASC-Ectos.

**Supplementary Information:**

The online version contains supplementary material available at 10.1186/s13287-024-03632-4.

## Background

Articular cartilage injury is common and can result in painful and disabling joint diseases in the long run [1]. It can be classified as a partial- or full-thickness cartilage defect, depending on the severity of the injury [2]. Cartilage has a limited ability to self-regenerate in the absence of vascular, neural, and lymphatic networks [3]. To date, non-steroidal anti-inflammatory drugs (NSAIDs) and intra-articular injections of hyaluronic acid (HA) are mainly used to alleviate symptoms in mild cases. Large-size cartilage defects are treated with surgical techniques such as microfracture, autologous chondrocyte implantation (ACI), matrix-assisted autologous chondrocyte implantation (MACI), and autologous osteochondral transplantation [4]. Nevertheless, the long-term clinical outcomes are unsatisfactory, as these treatments fail to produce hyaline cartilage and increase the incidence of delayed osteoarthritis. Therefore, new therapeutic modalities are urgently required for cartilage repair and regeneration.

As regenerative medicine advances, mesenchymal stromal cell (MSC)-based therapy offers a promising alternative for the repair of damaged or diseased cartilage tissues [5]. MSCs are capable of differentiating into chondrocytes in the area of a lesion and secreting cytokines and growth factors that facilitate cartilage regeneration [6]. There is increasing evidence to suggest that the clinical efficacy of MSCs is primarily related to their paracrine effects. The paracrine communication ability of extracellular vesicles (EVs) containing multiple therapeutic biomolecules has gained attention due to their ability to transfer their contents (nucleic acids, lipids, or proteins) to recipient cells [7]. Therefore, MSC-derived EVs (MSC-EVs) serve as a potential cell‐free therapy for cartilage regeneration, eliminating many difficult issues (e.g., immunogenicity and tumorigenicity) associated with using living cells as therapeutics. Furthermore, MSC-EVs can offer the potential advantages over MSCs of easy storage, transportation, and commercialization [8].

EVs are generally classified into different groups according to their biogenesis mechanisms, which include exosomes, ectosomes (also called microvesicles, shedding vesicles, or microparticles), and apoptotic bodies [9]. Exosomes are generated from intraluminal vesicles during the maturation of endosomes to multivesicular bodies (MVBs) and released by the fusion of the MVBs with the cell membrane [10]. Ectosomes and apoptotic bodies are directly shed from living cells and apoptotic cells, respectively, via outward budding of the plasma membrane [11]. A large body of research shows that MSC-derived exosomes (MSC-Exos) and ectosomes (MSC-Ectos) both can effectively reduce the production of inflammatory cytokines and apoptosis in osteoarthritic chondrocytes while increasing cartilage extracellular matrix (ECM) expression [12, 13]. Moreover, they can protect mice from developing osteoarthritis in vivo [14]. In addition, recent investigations have demonstrated that the combination of MSC-Exos and tissue engineering can augment cartilage defect regeneration in vivo [15–17]. However, MSC-Ectos have not been evaluated in an osteochondral defect model for cartilage repair. Moreover, the different biogenesis mechanisms mentioned above determine the dissimilarity among the two EVs concerning their morphological characteristics and cargo contents [18, 19]. Adipose-derived stem cells (ASCs) possess numerous desirable advantages, such as a less invasive collection process, high cellular yield, and great expansion capability [20]. In our prior study, we isolated exosomes and ectosomes from ASCs and analyzed their different micro-RNA expression profiles, though they partially overlap in size [21]. As we demonstrate, ASC-Exos have a stronger ability to inhibit inflammation than ASC-Ectos under the pathological condition of tendinopathy. Thus, it is valuable to determine whether the two EVs promote cartilage regeneration in similar or different manners under the pathological conditions of cartilage defects, as this information could lead to a deeper understanding of the role of EVs in vivo and provide guidance on their clinical use.

In this study, type I collagen hydrogel was employed as a delivery vehicle to optimize EV application in vivo in a model of the osteochondral defect and to prevent rapid clearance from the joint. The objective of this study was to compare the biological function of ASC-Exos with that of ASC-Ectos on chondrocytes and bone marrow-derived MSCs (BMSCs) in vitro and to verify whether ASC-EVs can enhance the reparative effect of the collagen hydrogel in a rat osteochondral defect model. Moreover, the in vivo effects of the two EVs on cartilage repair were compared, and the mechanisms underlying ASC-EV-mediated cartilage repair with respect to matrix synthesis, cell apoptosis, and macrophage response were investigated.

## Materials and methods

### Cell isolation and culture

Animal studies were reported in compliance with the ARRIVE guidelines (http://www.nc3rs.org.uk/page.asp?id=1357). The Animal Ethics Committee of the 2nd Affiliated Hospital, School of Medicine, Zhejiang University approved the animal research ethics. Isolation and culture of primary adipose-derived MSCs (ASCs) and chondrocytes from Sprague–Dawley rats were performed as we have described previously [21, 22]. Briefly, minced adipose tissue was digested with 0.1% collagenase type I and cartilage particles were digested with 0.2% collagenase II at 37 °C for 4 h. Next, collected cells were seeded into 25-mm^2^ culture flasks with corresponding mediums containing 10% fetal bovine serum (FBS). Rat bone marrow–derived MSCs (BMSCs) were purchased from OriCell (Cyagen, Guangzhou, China). All experiments were conducted with ASCs and BMSCs at the third passage (P3) and chondrocytes at the third to fifth passages (P3–P5). ASCs and BMSCs were cultured and expanded in MSC medium with 10% FBS (OriCell, Cyagen), while chondrocytes were cultured in DMEM/F12 medium with 10% FBS (Gibco, USA).

### Isolation of extracellular vesicles

Extracellular vesicles (EVs) were isolated by ultracentrifugation and characterized using nanoparticle tracking analysis (NTA), transmission electron microscope (TEM), and western blotting (WB) for protein maker detection, as described previously [21]. Briefly, ASCs were grown to 70% confluence and then incubated in DMEM/F12 medium containing 10% exosome-depleted FBS (pre-ultracentrifuged at 120,000 g overnight). After 48 h, conditioned media was collected and centrifuged at 300 g for 10 min, then 2000*g* for 20 min, and then filtered through a 0.8-μm filter (Millipore), followed by centrifugation at 16,500*g* for 45 min to isolate ectosomes. Exosomes were isolated by subsequent centrifugation at 110,000*g* for 70 min. The supernatant after pelleting the EVs was collected and defined as the "EV-depleted fraction". For in vitro and in vivo experiments, EV pellets were thoroughly washed once with phosphate-buffered saline (PBS) and resuspended in PBS and then stored at -80 °C until usage. All centrifugations were performed at 4 °C.

### EV uptake analysis

The EVs were labeled using a PKH26 red fluorescent labeling kit in accordance with the instructions provided by the manufacturer (Umibio Co. Ltd., Shanghai, China). Briefly, EVs were incubated with PKH26 dye for 10 min, rinsed with 10 ml PBS, and centrifuged at corresponding speeds for 45 min or 70 min. Following co-incubation with labeled EVs for 12 h in a 48-well plate, the chondrocytes and BMSCs were fixed in 4% paraformaldehyde (PFA) for 15 min and stained with 4′,6-Diamidino-2-phenylindole (DAPI) for 5 min. Fluorescence microscopy images were carried out by an inverted fluorescence microscope (Leica DMi8).

### Cell proliferation assays

The proliferation of cells was monitored using the CCK8 kit (Dojindo, Japan) as recommended by the manufacturer. In light of the scarcity of research concerning the utilization of ASC-Ectos in cell treatment, three concentrations of exosomes (1, 2, and 5 × 10^9^ particles/ml) were chosen for cell proliferation assessments, drawing upon previous investigations pertaining to ASCs and chondrocytes [23, 24]. The concentration of 2 × 10^9^ particles/ml (approximately equivalent to 20 μg/ml) was determined as the optimal choice for cell experiments based on the observed highest cell viability in the CCK8 assay. Six replicate wells of 5,000 cells per well were prepared in 96-well plates with BMSCs and chondrocytes. EVs were then added at a concentration of 2 × 10^9^ particles/ml, which was determined by NTA. At different time points (24 h, 48 h, and 72 h), 10 μl of CCK8 solution was added to each well and incubated for two hours. Then, absorbance at 450 nm was recorded using a microplate reader (SpectraMax® ABS, USA). Three replicates of the assay were performed.

### Migration assay

Transwell assays and scratch healing assays were used to evaluate cell migration. For the transwell assay, 2 × 10^4^ BMSCs and 5 × 10^4^ chondrocytes were seeded into the upper chamber of a transwell insert (8-μm pore) with serum-free medium, respectively. The inserts were then plated in the wells of 24-well plates containing 10% FBS medium with or without 2 × 10^9^/ml EVs. A 24-h incubation period was followed by the washing of the migrated cells using PBS, their fixation with 4% PFA for 15 min, and their staining with 0.05% crystal violet for 30 min. After removing the cells lining the upper surface of the membrane with cotton swabs, the migrated cells were observed under a microscope. At a magnification of 200 × , five randomly selected fields were photographed. In each field, positively stained cells were counted and calculated. For the scratch assay, the corresponding cells were cultured in 6-well plates until confluency, and cell wounds were created in each well using a 200-μl pipette. Following three rounds of washing with PBS, fresh medium with or without 2 × 10^9^ particles/ml EVs was added to the cells. Next, three images of each well were taken at 0 h, 24 h, and 48 h, and the wound area was measured using Image J (v.2.1.0). Each group was subjected to the experiment three times.

### Chondrocyte apoptosis assay

Chondrocyte apoptosis was measured using the Annexin V-APC/PI apoptosis kit (Multi Sciences, China). Chondrocytes were treated with 10 ng/ml of IL-1β and co-incubated for 24 h with equal concentrations of ectosomes and exosomes at a concentration of 2 × 10^9^ particles/ml. Cells of each group were collected in 500 μL binding buff and stained with 5 μL allophycocyanin (APC)-labeled Annexin V and 10 μL propidium iodide (PI) solution for 10 min in the dark at room temperature. Then, apoptotic cells were analyzed using a flow cytometer (Beckman Coulter, CytoFLEX LX) according to the manufacturer's instructions.

### Chondrogenic differentiation assay

BMSC chondrogenic differentiation was carried out using an OriCell Mesenchymal Stem Cell Chondrogenic Differentiation Kit (Cyagen, China) following the manufacturer's instructions. Briefly, 3 × 10^5^ BMSCs were transferred into 15-ml conical bottom tubes with 500 μl complete chondrogenic induction medium and centrifuged for 5 min at 1000 rpm. After cell pellets were formed (24–48 h), the bottom of the tube was gently bounced to suspend them in the medium, and the complete chondrogenic induction medium with or without 2 × 10^9^ particles/ml EVs was changed every 3 days for 21 days. The chondrogenic pellet was fixed in 4% PFA, dehydrated with 30% sucrose, sectioned at 10 μm, stained with Alcian blue solution, and photographed under a microscope.

### Reverse transcriptase quantitative RT polymerase chain reaction (qRT-PCR) assay

Total RNA was extracted from the cells using TRIzol (Invitrogen), and first-strand cDNA was synthesized from 0.5 μg RNA using the Transcriptor First Strand cDNA Synthesis Kit (Takala, Dalian, China) in accordance with the manufacturer's instructions. A qRT-PCR was performed with the SYBR Premix ExTaq kit (Takara, Dalian, China) to quantify the expression levels of mRNA encoding for the indicated genes. This reaction was conducted on an Applied Biosystems AB 7000 quantitative PCR machine at 95 °C for 5 min, 40 cycles of 95 °C for 5 s, and 60 °C for 30 s. Relative mRNA expression was calculated using the 2^−ΔΔCt^ method and normalized to the expression of β-actin. The following primer sequences were used: Col2a1 FWD- GGCCAGGATGCCCGAAAATTA, REV- CCCTCTCTCCCTTGTCACCAC, Aggrecan FWD- CTGGGTGGATGCAGAGAGAC, REV- TTGGTTTGGACGCCACTTCT, β-actin FWD- CACCTTCTACAATGAGCTGCGTGT, REV- CACAGCCTGGATAGCAACGTACA. Three independent experiments were conducted for each quantitative RT-PCR.

### WB analysis

Cell lysate was prepared by using RIPA buffer containing protease inhibitors and phosphatase inhibitors. Protein concentration was determined using a BCA protein assay kit, and the lysate was boiled for 20 min at 100 °C with a loading buffer. Equal amounts of protein extracts were separated on SDS-PAGE and transferred to polyvinylidene fluoride (PVDF) membranes (Millipore). Afterward, the PVDF membranes were blocked, incubated overnight at 4 °C with primary antibodies, and incubated with secondary antibodies for 1 h at room temperature. ECL reagent was used to detect immunoreactivity by a chemiluminescence detection system (GE Healthcare). The primary antibodies were anti-Aggrecan (1:200, NB600-504, NOVUS), anti-Collagen II (1:1000, ab34712, Abcam), and anti-β-actin (1:500, ab8226, Abcam).

### Surgical procedure

The osteochondral defect model was conducted using twenty-eight male Sprague–Dawley rats (8 weeks), randomly divided into the following groups (*n* = 7 in each group): control group (PBS), hydrogel group (Gel), hydrogel containing ectosomes group (Gel + Ectos), and hydrogel containing exosomes group (Gel + Exos). Following anesthesia with 2–4% isoflurane, the rats were depilated on the bilateral hind limb, and the skin was disinfected with iodophor. Next, an incision was made along the medial parapatellar margin to expose the articular capsules. The patella was also displaced slightly toward the medial condyle to expose the cartilage of the femoral patellar groove. A full-thickness cartilage defect (1.5 mm in diameter and 2 mm in depth) was created using a custom drill. At the same time, rat type I collagen hydrogels were prepared for embedding the EVs. Gelation of the hydrogels can be observed after a few minutes at 37 °C. To prevent premature gelation, reagents were kept on ice and a mixture of 40 μl type I collagen (5 mg/ml, Corning 354,236); 2.4 μl NaOH (0.1 M, Sigma 655,104); 20 μl PBS 10X (Sigma D5523); and 37.6 μl EV solution was prepared. The defects in each experimental group were implanted with corresponding hydrogels, and the control group was treated with PBS. In order to fill the defect area adequately, it is imperative to inject 10 μl of type I collagen gel into the osteochondral defect, resulting in an approximate final EV concentration of 7.5 × 10^9^ particles. Should the concentration surpass this threshold, it may impede the effective re-suspension of EVs or hinder the anticipated formation of the hydrogel within the designated timeframe. Following the solidification of the hydrogel at 37 °C, a suture was interrupted to close the joint, and animals were allowed free-cage activity.

### Postoperative assessment

#### Macroscopic evaluation

At 4 and 8 weeks after surgery, rats were euthanized with an overdose of pentobarbital, and each distal femur was isolated, exposed, and photographed to obtain macrographs for assessment by three independent surgeons following the International Cartilage Repair Society (ICRS) scoring system (Additional file 7: Table s1) [25].

#### Histology

The femur was explanted from the exposed joint cavity and fixed with 4% PFA for 48 h. The harvested osteochondral specimens were first prepared for micro-computed tomography (micro-CT) analysis and then decalcified in 10% ethylenediaminetetraacetic acid (EDTA) for 4 weeks with daily changes of the decalcification solution. The specimens were then cut in half through the center of the defect along the longitudinal axis of the femoral patellar groove. After being embedded in paraffin, sectioned (5-μm sections), de-paraffinized, and re-hydrated, the tissues were prepared for histology. Staining with hematoxylin and eosin (HE), Safranin O/fast green, Alcian blue, and Sirius red was performed according to standard procedures. Quantitative assessment of cartilage repair was conducted by three independent surgeons using the modified O'Driscoll histologic score (Additional file 8: Table s2) [26].

#### Immunohistochemistry

For immunohistochemistry staining, sections were subjected to heat-mediated antigen retrieval using citrate (pH 6.0), blocked in 10% horse serum, and then incubated in primary antibodies overnight at 4 °C. After being incubated with biotinylated secondary antibodies for 1 h at room temperature, sections were exposed to diaminobenzidine (DAB) to complete the color reaction. The following primary antibodies were used for immunohistochemistry: anti-Collagen I (1:100, ab34710, Abcam), anti-Collagen II (1:200, ab34712, Abcam), anti-CD206 (1:2000, ab64693, Abcam), and anti-Arginase-1 (1:500, #93,668. CST).

#### In vivo apoptosis analysis

Articular chondrocyte apoptosis was determined by the TUNEL (terminal deoxynucleotidyl transferase dUTP nick-end labeling) assay performed using the TUNEL Apoptosis Detection Kit (Millipore). All images were taken using suitable microscopes. The optical density quantification of immunohistochemical images was conducted using Image-Pro Plus 6.0 software, and the average optical density was determined. In addition, the proportion of apoptotic articular chondrocytes to the total number of articular chondrocytes was measured.

#### Micro-CT analysis

The reconstructed subchondral bone in the osteochondral defect of each group (*n* = 6) was assessed using a SkyScan 1272 micro-CT scanner (Bruker Micro CT, Kontich, Belgium). The scanning parameters used were as follows: voltage = 70 kV, voxel resolution = 18 μm, aluminum filter = 0.2 mm, and current = 112 μA. Reconstructions of the scan images were performed using three-dimensional model visualization software (CTVol). A fixed threshold (1400) was used to extract the mineralized bone phase, and trabecular bone volume fraction (BV/TV), bone mineral density (BMD), and trabecular separation (Tb.Sp) were quantitatively determined to assess the healing quality of subchondral bone.

### Proteomics

The protein extraction, SDS-PAGE electrophoresis, FASP enzymatic hydrolysis, and mass spectrometry (MS) were performed by Shanghai Jikai Gene Chemical Technology Co., Ltd. Briefly, SDT buffer (4%SDS, 100 mM Tris–HCl, pH 7.6) was used for protein extraction, and 20 µg of proteins for each sample was separated on 12% SDS-PAGE gel. Then, protein bands were visualized by Coomassie Blue R-250 staining. Next, 50 ug of proteins were reduced, washed, and digested by repeated ultrafiltration (Sartorius, 30kD). Following desalination of the resulting peptide segment by C18 column, 2 μg of peptide was used for MS. MS analysis was performed on a Orbitrap Exploris 480 mass spectrometer (Thermo Fisher Scientific) that was coupled to Easy nLC (Thermo Fisher Scientific). The MS data were analyzed using MaxQuant software version 1.6.17.0. Proteins which Fold change > 1.2 and p value (Student’s t test) < 0.05 were considered to be a differentially expressed protein. Based on the Fisher's exact test, we compared the distribution of each GO term or KEGG pathway in the target protein set.

### Statistical analysis

The size of the sample for the animal experiment was determined based on previous experience [27, 28]. Data were analyzed with GraphPad Prism version 9.1.1 (GraphPad Software, San Diego, CA). Shapiro–Wilk testing was performed on all data in the first step to ensure that they met the normality distribution (all passed). One-way analysis of variance (ANOVA) was used to analyze the data, followed by Tukey's post hoc test for pairwise comparisons. Values are expressed as the mean ± standard error (SD), and a p-value < 0.05 was considered to indicate a statistically significant difference between two groups.

## Results

### Isolation and characterization of ASC-Ectos and ASC-Exos

Additional file 1: Fig. S1A illustrates the procedure for isolating EVs derived from ASCs using differential ultracentrifugation. The TEM and NTA results were generally consistent with those of our previous study, in which the WB results showed that ASC-Exos expressed exosomal markers CD9, CD63, Tsg101, Hsp70, and Alix, while ASC-Ectos expressed CD63 and Alix [21]. TEM analysis revealed that both EVs had a round, had a spherical shape and were composed of double-layered membranes (Additional file 1: Fig. S1B). NTA analysis revealed that the mean particle sizes of ASC-Exos and ASC-Ectos were 146.9 ± 13.3 nm and 250.8 ± 1.9 nm, respectively (Additional file 1: Fig. S1C). However, there is a partial overlap in the particle sizes between the two EV subpopulations, indicating that each vesicle population contains some components of the other. Further, the protein expression of the 2 EVs was analyzed by Proteomics, as described later.

### Effects of ASC-Ectos and ASC-Exos on proliferation, migration, and apoptosis of chondrocytes

**Fig. 1:**
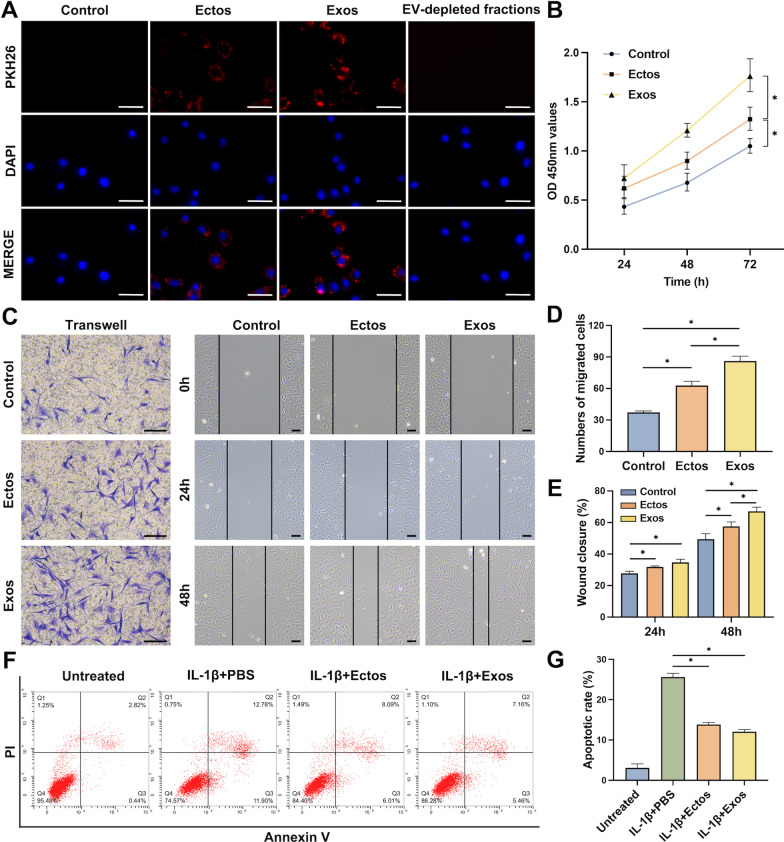
Effect of ASC-Ectos and ASC-Exos on the proliferation, migration, and apoptosis of chondrocytes. PBS treatment served as a control. (**a**) Representative fluorescence micrograph of EV uptake by chondrocytes. The blue and red colors represent the nucleus and PKH26-labeled EVs, respectively. (**b**) Chondrocyte viability measured by CCK-8 at 24, 48, and 72 h after co-incubation with EVs. (**c**) Representative images showing the transwell migration assay and scratch assay of chondrocytes treated with EVs for 24 and 48 h. (**d**) Quantitative analysis of the migrated chondrocytes. (**e**) Quantitative analysis of the wound closure. (**f**) Flow cytometry analysis of apoptosis in chondrocytes with different treatments. (**g**) Quantitative evaluation of chondrocyte apoptotic rate. Scale bar: 100 μm. ^*^p < 0.05. Data were presented as mean ± SD of three replicates

To investigate ASC-EV uptake by chondrocytes, PKH26-labeled EVs and EV-depleted fractions were incubated with chondrocytes for 12 h. In fluorescence microscopy images (Fig. 1a), PKH26-labeled EVs (red fluorescence) were visible surrounding chondrocyte nuclei (blue fluorescence stained with DAPI). The results showed ingestion of both ASC-Ectos and ASC-Exos by chondrocytes, potentially influencing chondrocyte proliferation, migration, and apoptosis. As a next step, we examined cell proliferation by using the CCK8 assay. As compared to the control group, ASC-EVs stimulated chondrocyte proliferation, but ASC-Exos was more effective (Fig. 1b). An assessment of chondrocyte migration in different treatment groups was conducted using the transwell and scratch wound assays. The migrated chondrocytes and scratch area were visible in the light microscopic photographs (Fig. 1c). Statistical analysis revealed significant enhancement of chondrocyte motility by both ASC-Ectos and ASC-Exos, with ASC-Exos outperforming ASC-Ectos at equivalent concentrations (Fig. 1d, e). Annexin V staining followed by flow analysis showed increased apoptosis in IL-1β induced chondrocytes compared with the untreated cells. Moreover, both ASC-Ectos and ASC-Exos significantly reduced apoptosis, with no statistical differences between the two EV groups (Fig. 1f, g).

### Effects of ASC-Ectos and ASC-Exos on proliferation, migration, and chondrogenesis of BMSCs

**Fig. 2:**
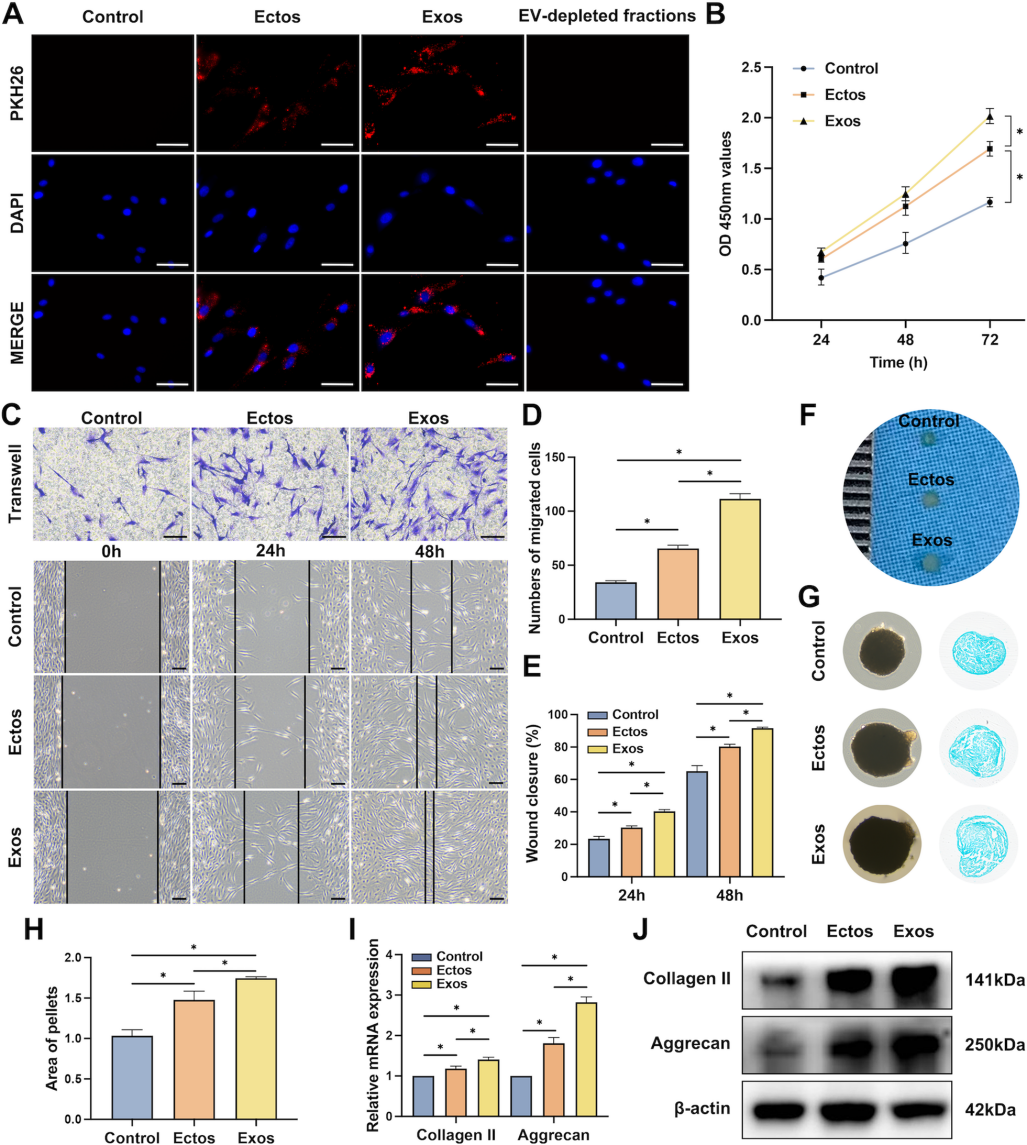
Effect of ASC-Ectos and ASC-Exos on the proliferation, migration, and chondrogenesis of BMSCs. PBS treatment served as a control. (**a**) Representative fluorescence micrograph of EV uptake by BMSCs. The blue and red colors represent the nucleus and PKH26-labeled EVs, respectively. (**b**) BMSC viability measured by CCK-8 at 24, 48, and 72 h after co-incubation with EVs. (**c**) Representative images showing the transwell migration assay and scratch assay of BMSCs treated with EVs for 24 and 48 h. (**d**) Quantitative analysis of the migrated BMSCs. (**e**) Quantitative analysis of the wound closure. (**f**) Representative micrograph of BMSC chondrogenic microspheres. (**g**) Representative light micrograph (left) and Alcian blue staining (right) of BMSC chondrogenic microspheres. (**h**) Quantitative analysis of chondrogenic microsphere pellet area. (**i**) Gene expression level of collagen II and aggrecan detected by qPCR. (**j**) Protein expression level of collagen II and aggrecan detected by WB. Scale bar: 100 μm. ^*^p < 0.05. Data were presented as mean ± SD of three replicates

Similarly, PKH26-labeled EVs and EV-depleted fractions were incubated with BMSCs to determine the uptake of ASC-EVs by BMSCs. As a result of co-culturing for 12 h, the distribution of ASC-Ectos and ASC-Exos appeared particularly distinct around the nucleus, indicating that the two EVs were gradually internalized by BMSCs (Fig. 2a). According to the CCK8 assay results, both ASC-Ectos and ASC-Exos significantly enhanced the proliferation potential of BMSCs, while ASC-Exos had the greatest effect on viability (Fig. 2b). The migration of BMSCs was assessed using transwell and scratch assays with or without ASC-Ectos or ASC-Exos. At 24 and 48 h post-scratching, both groups exhibited accelerated wound healing compared to the control, with ASC-Exos showing faster recovery than ASC-Ectos. Consistently, ASC-EV treatment enhanced BMSC migration in transwell assays, with ASC-Exos outperforming ASC-Ectos at the same concentration (Fig. 2c-e). Additionally, we sought to confirm the differentiation ability of BMSCs with ASC-EV treatment. After 21 days of chondrogenesis, the macroscopy and light microscopic photographs showed that the cell microspheres treated with ASC-EVs were significantly larger than the control group (Fig. 2f, g). The differentiation of BMSCs into chondrocytes was confirmed by the detection of glycosaminoglycan deposits using Alcian blue staining. Statistical analysis revealed that the cross-sectional area of microspheres in the ASC-Exo group was greater than that in the ASC-Ecto group at the same concentration (Fig. 2h). Consistently, ASC-EV treatment significantly increased the mRNA and protein expression levels of chondrogenesis-related genes such as type II Collagen and Aggrecan in BMSCs, as revealed by the PCR and WB results (Fig. 2i, j). Therefore, ASC-EVs were conducive to glycosaminoglycan formation and type II collagen deposition in BMSCs. Moreover, ASC-Exos were more effective than ASC-Ectos at promoting chondrogenic differentiation of BMSCs at the same concentration.

### In vivo macroscopic and histomorphological evaluation of repaired cartilage treated with ASC-Ectos and ASC-Exos

**Fig. 3:**
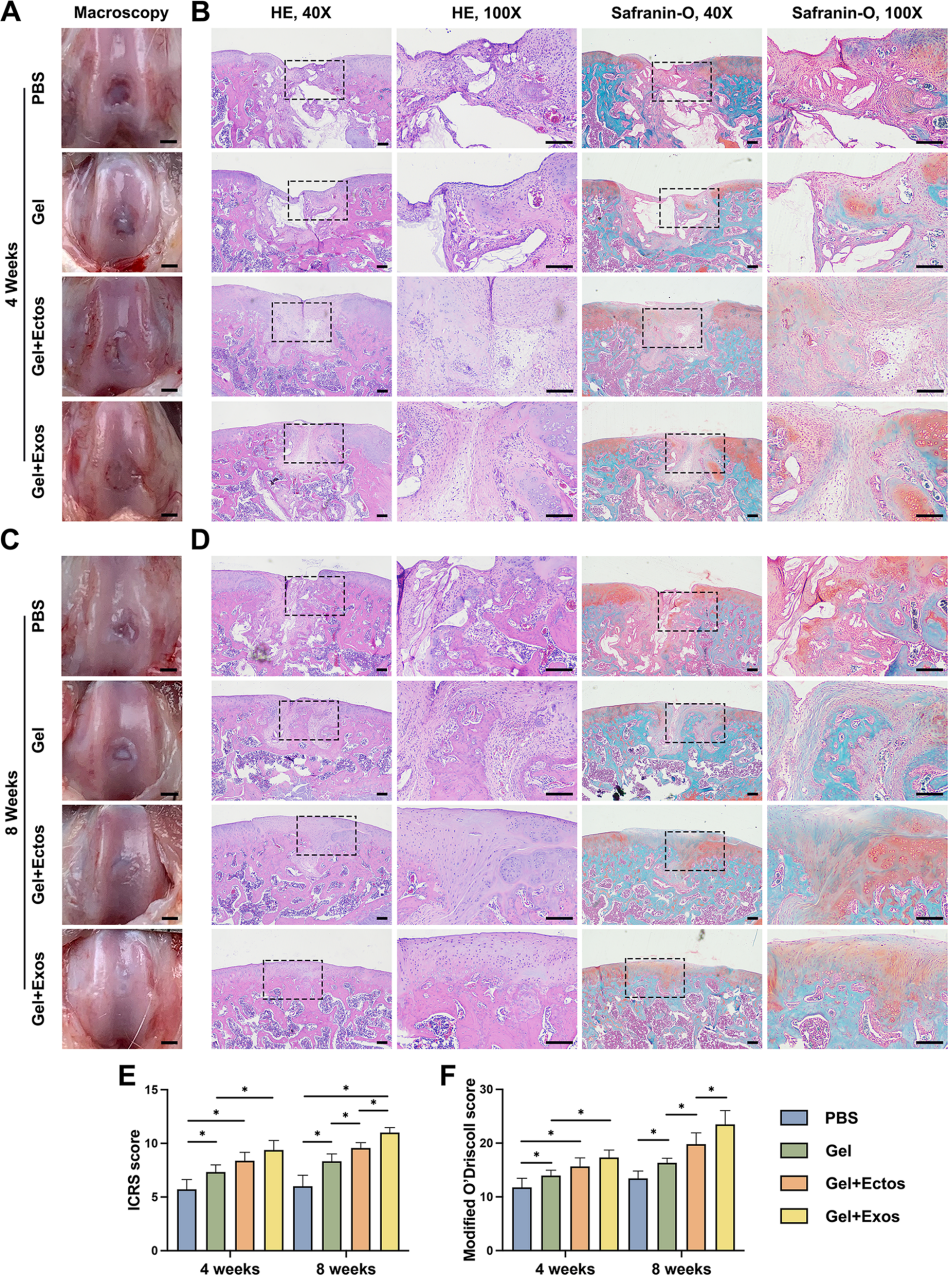
Assessment of cartilage repair in vivo. (**a**) Macroscopic observation of cartilage defects at 4 weeks. Scale bar: 2 mm. (**b**) HE and Safranin O/fast green staining of repaired cartilage at 4 weeks. Scale bar: 500 μm. (**c**) Macroscopic observation of cartilage defects at 8 weeks. Scale bar: 2 mm. (**d**) HE and Safranin O/fast green staining of repaired cartilage at 8 weeks. Scale bar: 500 μm. (**e**) ICRS macroscopic scores of different groups. (**f**) Modified O’Driscoll scores of different groups. ^*^p < 0.05. Data were presented as mean ± SD of seven replicates

For the local application of ASC-EVs in cartilage defects, we integrated ASC-EVs with type I collagen hydrogel in order to optimize in vivo application and ensure sustained release. The macroscopic and histomorphological results of cartilage regeneration at 4 weeks after surgery are shown in Fig. 3a, b. Macroscopically, neotissue was observed at the defect site in all groups. In the PBS group, limited fibrous tissue with a rough surface was visible, and defects beyond cartilage thickness were evident, showing a significant height difference and an uneven surface. Although the Gel group's repaired tissue did not fully fill the defect, it had a larger repair area and greater height than the PBS group. Moreover, the defect area still appeared to have residual collagen hydrogel and had not been completely replaced by neotissue, suggesting that Type I collagen hydrogel can persist for several weeks. The Gel + Ecto and Gel + Exo groups essentially filled the defect, but cracks persisted in Gel + Ecto, and boundaries were visible in both. HE staining aligned with macroscopic findings. PBS and Gel groups had disorderly fibrous tissue with minimal subchondral bone reconstruction. Gel + Ecto and Gel + Exo groups showed accelerated bone reconstruction, with more compact and organized repair tissue. However, a visible boundary remained. Safranin O/fast green staining indicated cartilage differentiation initiation, absent in PBS and partially present in the other groups, suggesting ongoing matrix secretion at this treatment stage.

At 8 weeks after surgery, the filling ratio of repaired tissue in the defect area was greater than that at four weeks, as observed macroscopically, with almost no visible residual collagen hydrogel (Fig. 3c). In the PBS group, the repaired tissue remained lower than adjacent cartilage, with an uneven surface. The other groups exhibited similar heights between repaired tissue and adjacent cartilage. Gel + Ecto and Gel + Exo groups showed cartilage-like neotissue with smooth surfaces, while the Gel group had fibrous tissue-like neotissue. Additionally, the boundary between repaired tissue and adjacent cartilage was less distinct in the Gel + Exo group compared to Gel + Ecto. Consistently, the histological result of the PBS group was the worst (Fig. 3d). In the PBS and Gel groups, fibrous repair tissues dominated, with limited positive Safranin O/fast green staining. However, the Gel group exhibited improved subchondral bone remodeling and fewer gaps than the PBS group. Gel + Ecto and Gel + Exo groups displayed cartilage-like neotissue with distinct cartilage lacuna, but the Gel + Exo group demonstrated more uniform and mature repaired cartilage. Notably, Safranin O/fast green staining revealed increased red staining, indicating accelerated cartilage repair and higher proteoglycan content in the Gel + Exo group compared to the Gel + Ecto group. Combined with the in vitro results, the sustained 8-week reparative effects may stem from enhanced BMSC and chondrocyte activities in the defect area, boosting matrix synthesis, aiding cartilage repair despite halted EV release.

The quantitative evaluation results of cartilage repair were analyzed by ICRS macroscopic score and modified O’Driscoll score (Additional file 9: Table s3). The ICRS macroscopic score showed significantly worse repair in the PBS group than in the other three groups at 4 and 8 weeks (Fig. 3e). In the Gel + Exo group, the macroscopic scores were higher compared to those of the Gel group at 4 weeks and higher compared to those of the Gel and Gel + Ecto groups at 8 weeks, which was consistent with the histological score results (Fig. 3f). The data suggest that ASC-EVs presented a favorable stimulatory effect for cartilage regeneration, whereas ASC-Ecto performed better at the same concentration over time. Since type I collagen hydrogels have been extensively studied and applied in cartilage defect repair, including basic research in various animals and clinical trials in humans, we did not quantify their degradation in our study.

### Micro-CT evaluation of subchondral bone reconstruction in vivo

**Fig. 4:**
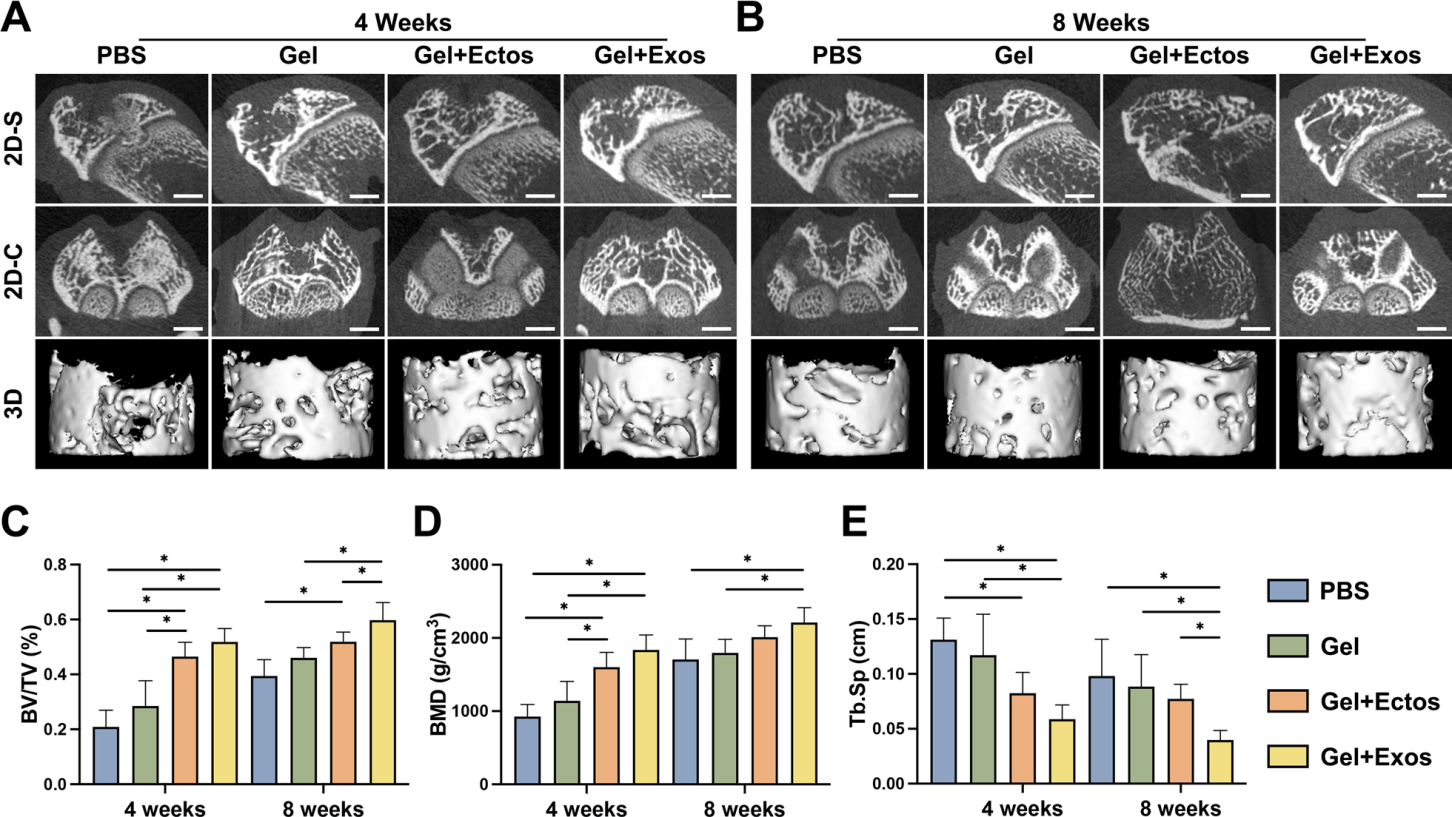
Micro-CT observation of subchondral bone regeneration. (**a**) Micro-CT images of different groups in different planes at 4 weeks. (**b**) Micro-CT images of different groups in different planes at 8 weeks. (**c-e**) Quantitation of bone volume to total volume (BV/TV), bone mineral density (BMD), and trabecular separation (Tb.Sp). Scale bar: 2 mm. ^*^p < 0.05. Six individual samples in each group were analyzed. Data were presented as mean ± SD of six biological replicates

Micro-CT was performed to evaluate the bony remodeling in the subchondral bone under the cartilage lesion. The representative micro-CT images from all groups showed varying degrees of bone defects at 4 weeks after surgery, and the Gel + Ecto and Gel + Exo groups displayed comparably small defects (Fig. 4a). At 8 weeks after surgery, the subchondral bone had been fully reconstructed in the Gel + Exo group (Fig. 4b). However, there were small bone defects visible in the Gel + Ecto group with a better reconstruction effect than in the Gel group. Quantitative analysis showed that BV/TV, BMD, and Tb.Sp of the Gel + Ecto group were comparable to the Gel + Exo group and significantly increased compared to the PBS group at 4 weeks, as shown in Fig. 4c-e. Additionally, BV/TV in the Gel + Exo group was significantly higher than that in the Gel + Ecto group, whereas Tb.Sp in the Gel + Exo group was significantly lower than that in the Gel + Ecto group at 8 weeks. The results indicated that ASC-EVs are capable of promoting subchondral bone reconstruction over time and that ASC-Exos have a greater capacity for this than ASC-Ectos.

### Evaluation of cartilage matrix remodeling and collagen fibril arrangement

**Fig. 5:**
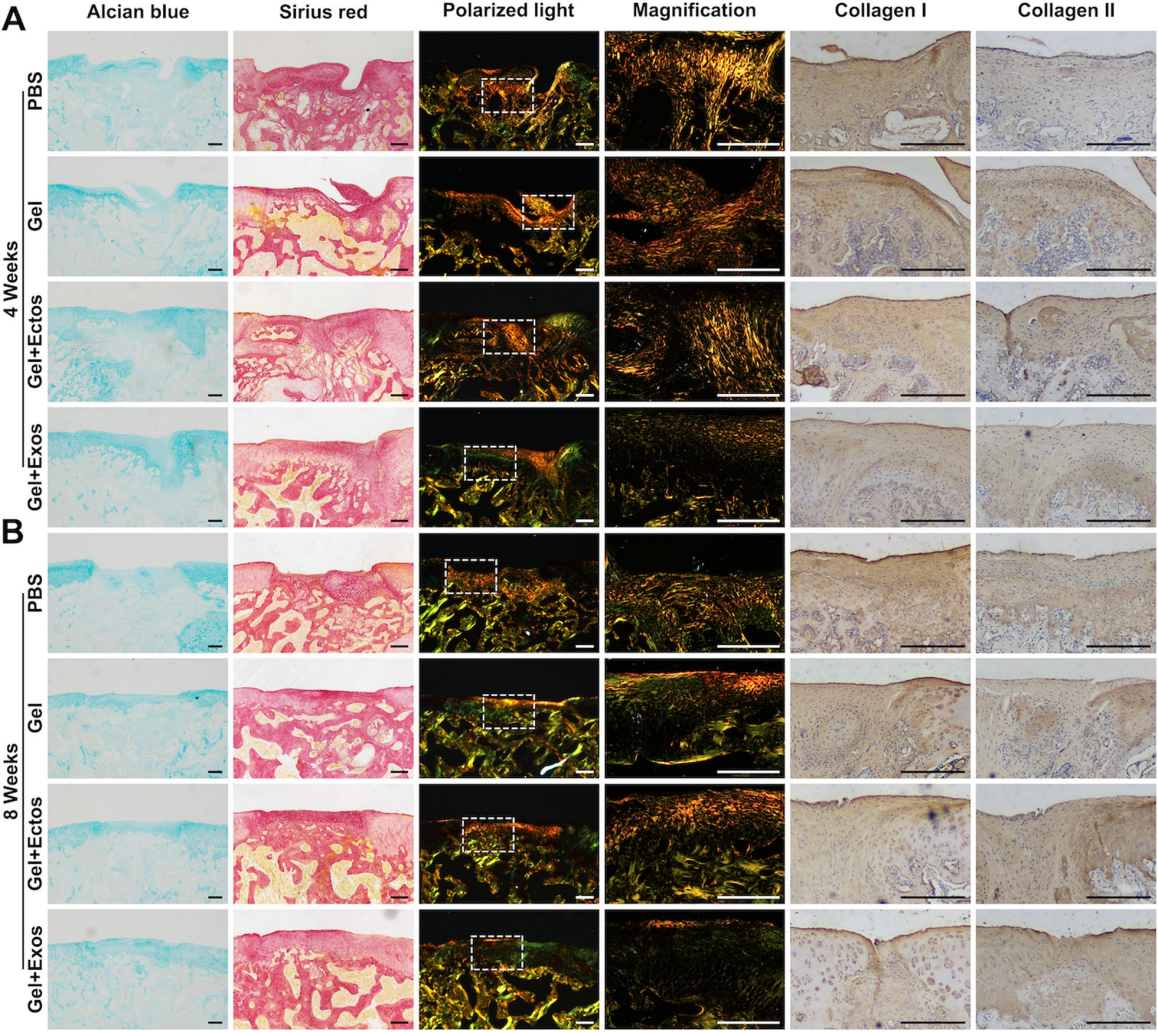
Cartilage matrix and collagen fibril observation. (**a**) Alcian blue staining of cartilage proteoglycans, Sirius red staining of collagen fibrils, polarized light images, and immunohistochemical staining of type I and type II collagen at 4 weeks. (**b**) Alcian blue staining of cartilage proteoglycans, Sirius red staining of collagen fibrils, polarized light images, and immunohistochemical staining of type I and type II collagen at 8 weeks. Scale bar: 200 μm. Six individual samples in each group were analyzed

At 4 weeks after surgery, Alcian blue staining showed that the blue cartilage matrix (hyaluronic acid and acid mucin) was more intense in the Gel + Ecto and Gel + Exo groups than in the PBS and Gel groups (Fig. 5a). Additionally, in the Gel + Exo group, the positive staining area at the repaired site appeared larger than that in the Gel + Ecto group. Sirius red staining under polarized light revealed type II collagen with weak birefringence (yellowish-green) and type I collagen with strong birefringence (reddish-yellow). Collagen fibers in the PBS and Gel groups were primarily type I collagen, arranged irregularly. Gel + Ecto and Gel + Exo groups showed more regular type I collagen arrangement. Notably, only the Gel + Exo group exhibited perpendicular alignment of type II collagen fibers in the middle layer. Immunohistochemical staining corroborated these findings, with the Gel + Exo group showing the lowest expression of type I collagen and the PBS group displaying the lowest expression of type II collagen. Although type II collagen expression was similar in all groups except PBS, Gel + Exo had a distinct distribution in the middle layer, as confirmed by polarized light.

At 8 weeks after surgery, Alcian blue staining demonstrated evenly distributed blue matrices as compared with the 4-week cartilage in all groups except the PBS group (Fig. 5b). However, the blue cartilage matrix in Gel + Ecto and Gel + Exo groups was thinner at 8 weeks than at 4 weeks due to incomplete subchondral bone reconstruction in the 4-week sections, with the defect still filled with fiber-like or cartilage-like neotissue. Notably, the blue staining in the Gel + Exo group closely resembled that of adjacent normal cartilage, surpassing the Gel + Ecto group. Polarized light revealed Gel + Exo's cartilage repair sites primarily composed of type II collagen, with type I collagen present only in the superficial region. The arrangement became more regular, with deep-layer type II collagen oriented vertically to the surface, anchoring to the subchondral bone plate. In contrast, the Gel + Ecto group had concentrated superficial and intermediate type I collagen, more regularly arranged along the surface than the Gel group. The Gel group exhibited a small amount of type II collagen, while none was detected in the PBS group. Immunohistochemistry confirmed differences in collagen deposition across all groups, consistent with the described results.

### Effects of ASC-Ectos and ASC-Exos on apoptosis during cartilage regeneration

**Fig. 6:**
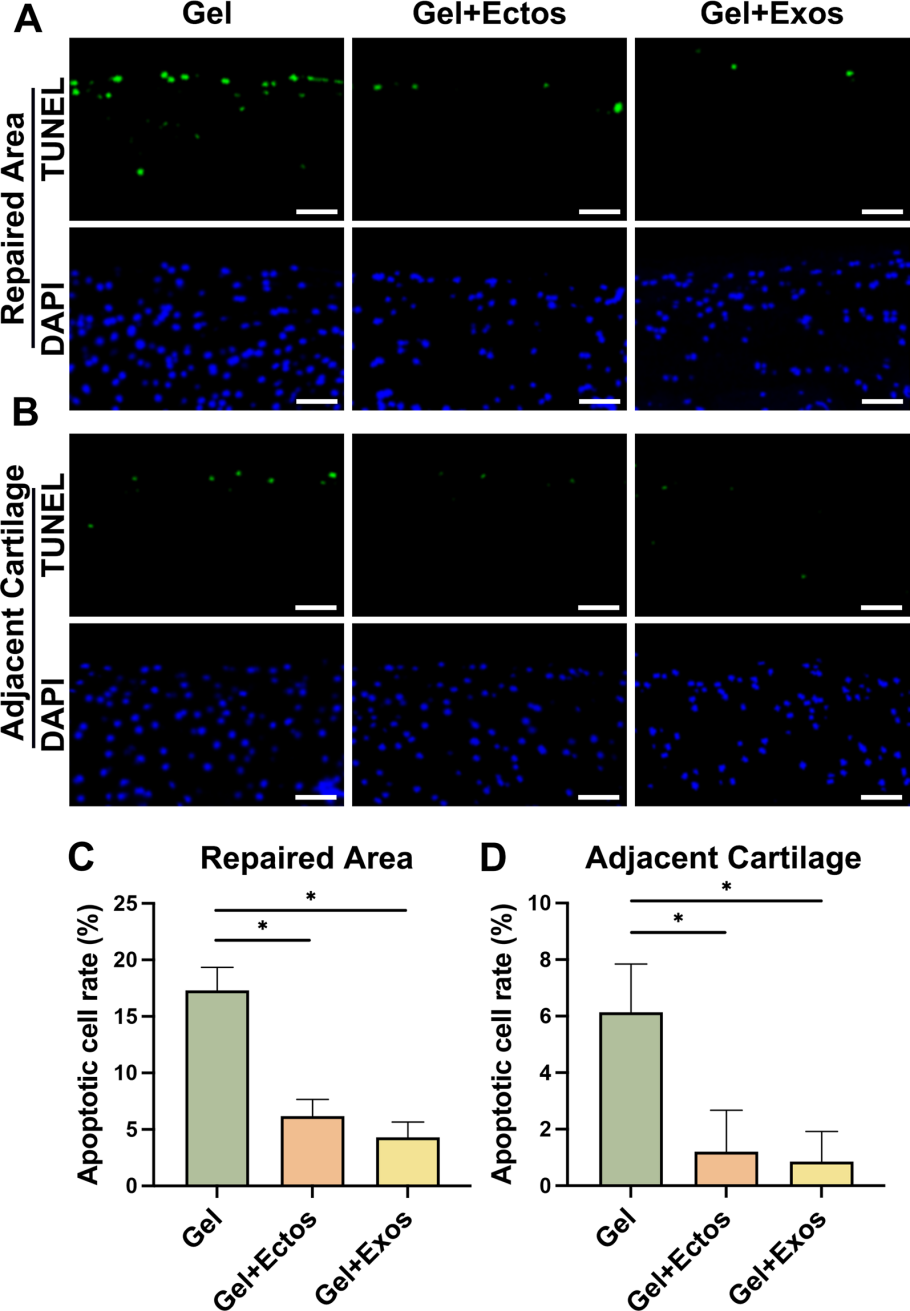
Effects of ASC-Ectos and ASC-Exos on apoptosis at 8 weeks of cartilage regeneration. (**a**) TUNEL staining of apoptotic cells in the repaired cartilage. (**b**) TUNEL staining of apoptotic cells in the adjacent cartilage. (**c**) Quantitative analysis of the cell apoptosis in the repaired cartilage. (**d**) Quantitative analysis of the cell apoptosis in the adjacent cartilage. Scale bar: 200 μm. ^*^p < 0.05. Data were presented as mean ± SD of six replicates

The chondrocyte apoptosis contributes significantly to cartilage degeneration following injury. Based on the above in vivo results, we found that the cartilage repair effect of the other three groups was superior to that of the PBS group. To determine the effects of ectosomes and exosomes on apoptosis in vivo, TUNEL staining was performed to detect apoptosis of chondrocytes in the repaired neotissue and adjacent cartilage of Gel, Gel + Ecto, and Gel + Exo groups at 8 weeks following cartilage regeneration (Fig. 6a, b). Four-week specimens were excluded due to the small number of chondrocytes in the repaired sites. In the Gel + Ecto and Gel + Exo groups, TUNEL staining showed a decrease in cell apoptosis of the repaired area compared to the Gel group (Fig. 6c). As in the repaired neotissue, adjacent cartilage regions of the Gel group showed an increase in chondrocyte apoptosis, and ASC-EV treatment inhibited chondrocyte apoptosis (Fig. 6d). The results indicate that ASC-EVs inhibited chondrocyte apoptosis in vivo, thereby improving cartilage repair and reducing cartilage destruction after trauma.

### Effects of ASC-Ectos and ASC-Exos on M2 macrophage infiltration during cartilage regeneration

**Fig. 7:**
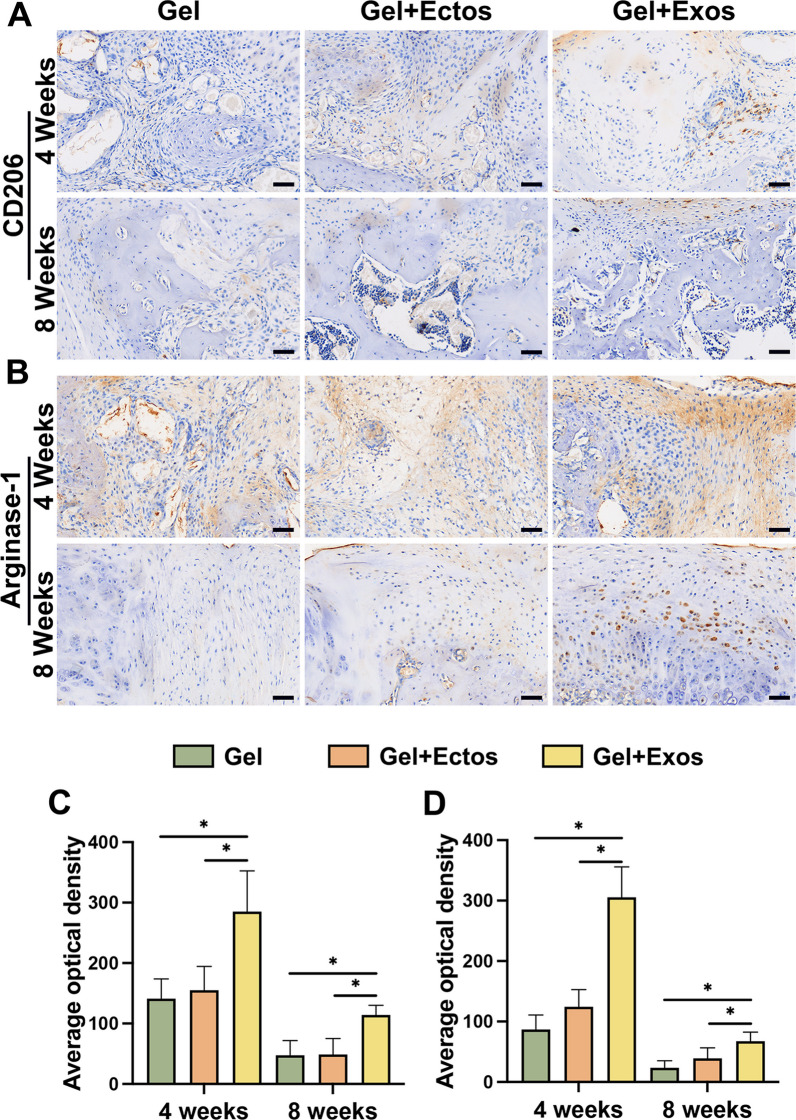
Effects of ASC-Ectos and ASC-Exos on M2 macrophage infiltration during cartilage regeneration. (**a**) Immunohistochemical staining of CD206 in different groups at 4 and 8 weeks. (**b**) Immunohistochemical staining of Arginase-1 in different groups at 4 and 8 weeks. (**c**) Quantitative analysis of CD206 protein expression by average optical density values. (**d**) Quantitative analysis of Arginase-1 protein expression by average optical density values. Scale bar: 50 μm. ^*^p < 0.05. Data were presented as mean ± SD of six replicates

The immune response to tissue injury and repair is an integral component of the healing process, so we next investigated whether ASC-EVs express immunomodulatory properties during their reparative activity. Specifically, we evaluated whether M2 macrophage infiltration into exosome-treated and ectosome-treated defects differed. Within 4 weeks of repairing the tissue, we observed the presence of numerous M2 macrophages, as evidenced by CD206 and Arginase-1 (Fig. 7a, b). The positive staining was more extensive in the Gel + Exo group than in the Gel and Gel + Ecto groups. Thereafter, the positive markers of M2 macrophages declined in all groups, but those in the Gel + Exo group remained consistently higher in number than those in the other groups. From the quantitative results, no significant difference was observed between the Gel and Gel + Ecto groups despite the Gel + Ecto group still showing higher expression of CD206 and Arginase-1 than the Gel group (Fig. 7c, d). Furthermore, the CD206 average optical density of the Gel + Exo group displayed a significantly higher value compared with that of the Gel and Gel + Ecto groups at both 4 weeks and 8 weeks. The increased CD206 expression in the Gel + Exo group was consistent with the significantly higher levels of Arginase-1.

### Proteomic analysis of ASC-Ectos and ASC-Exos

**Fig. 8:**
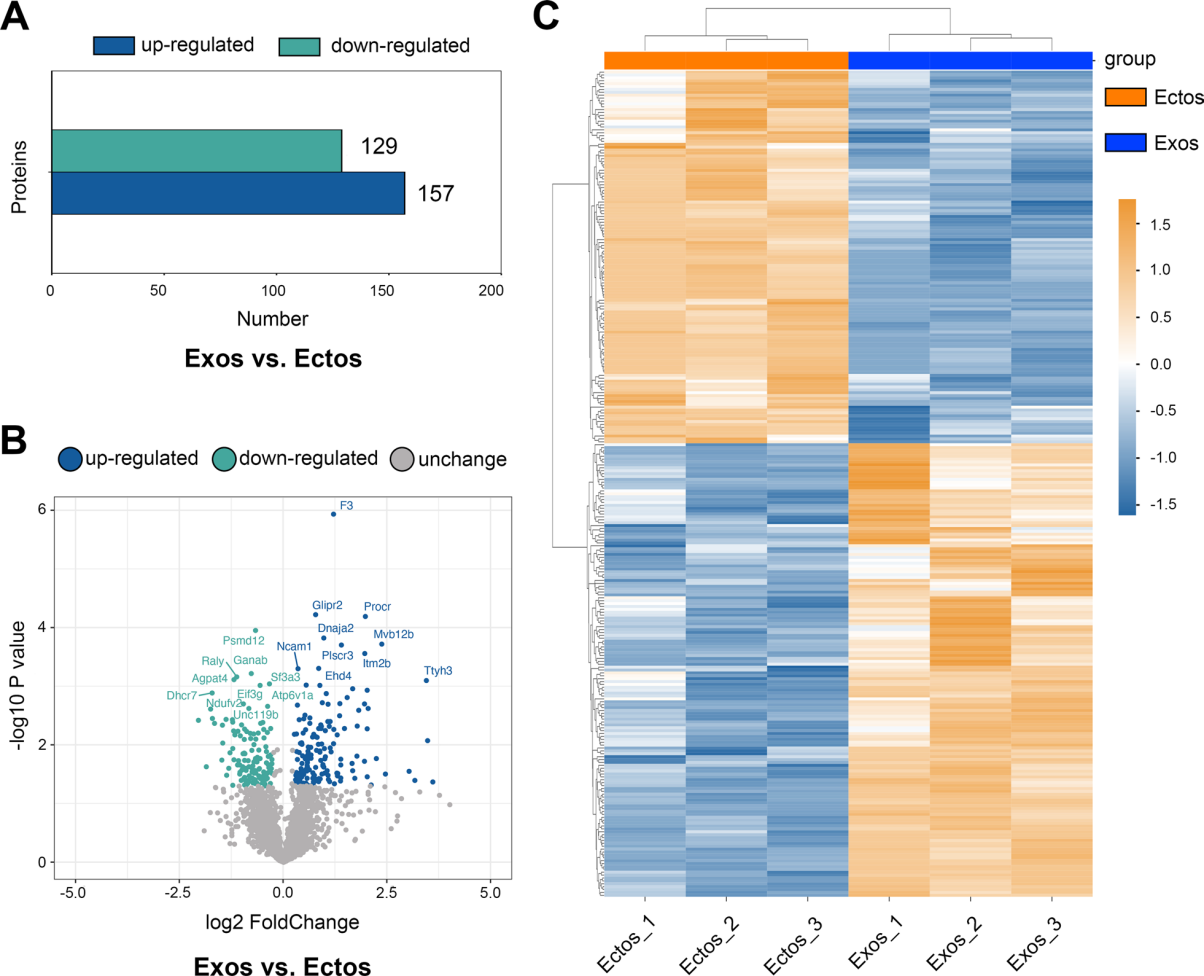
Proteomic analysis of ASC-Ectos and ASC-Exos. (**a**) Column graphs display the differential expression of proteins between ASC-Exos and ASC-Ectos. (**b**) The volcano plot displays the overall distribution of differentially expressed proteins. (**c**) Heat map showing the difference between ASC-Exo and ASC-Ecto protein profiles

Our previous study identified the different miRNA profiles of ASC-Ectos and ASC-Exos. Here, we analyzed the differences in protein expression, another crucial functional molecule in ASC-EVs. The number of differential proteins between ASC-Exos and ASC-Ectos is shown in a histogram (Fig. 8a), and a volcano plot was constructed to display the differential protein distribution between the two groups (Fig. 8b). Top 9 up-regulated and down-up-regulated proteins are shown in Additional file 2: Fig. S2 and Additional file 3: Fig. S3, respectively. To present our data more clearly and attractively, the cluster analysis was performed and then visualized by using a heat map (Fig. 8c). The differential proteins were functionally annotated using GO and KEGG, and the results of the top 10 GO enrichment categories in each category, as well as the top 20 KEGG pathway enrichment categories, are displayed in the bar chart (Additional file 4: Fig. S4 and Additional file 5: Fig. S5). Protein–protein interactions (PPI) of the targets were analyzed using the PPI network (Additional file 6: Fig. S6). Overall, these results indicate that the difference in protein profiling could influence the effects on cartilage regeneration when the 2 EV subtypes are administered.

## Discussion

**Fig. 9:**
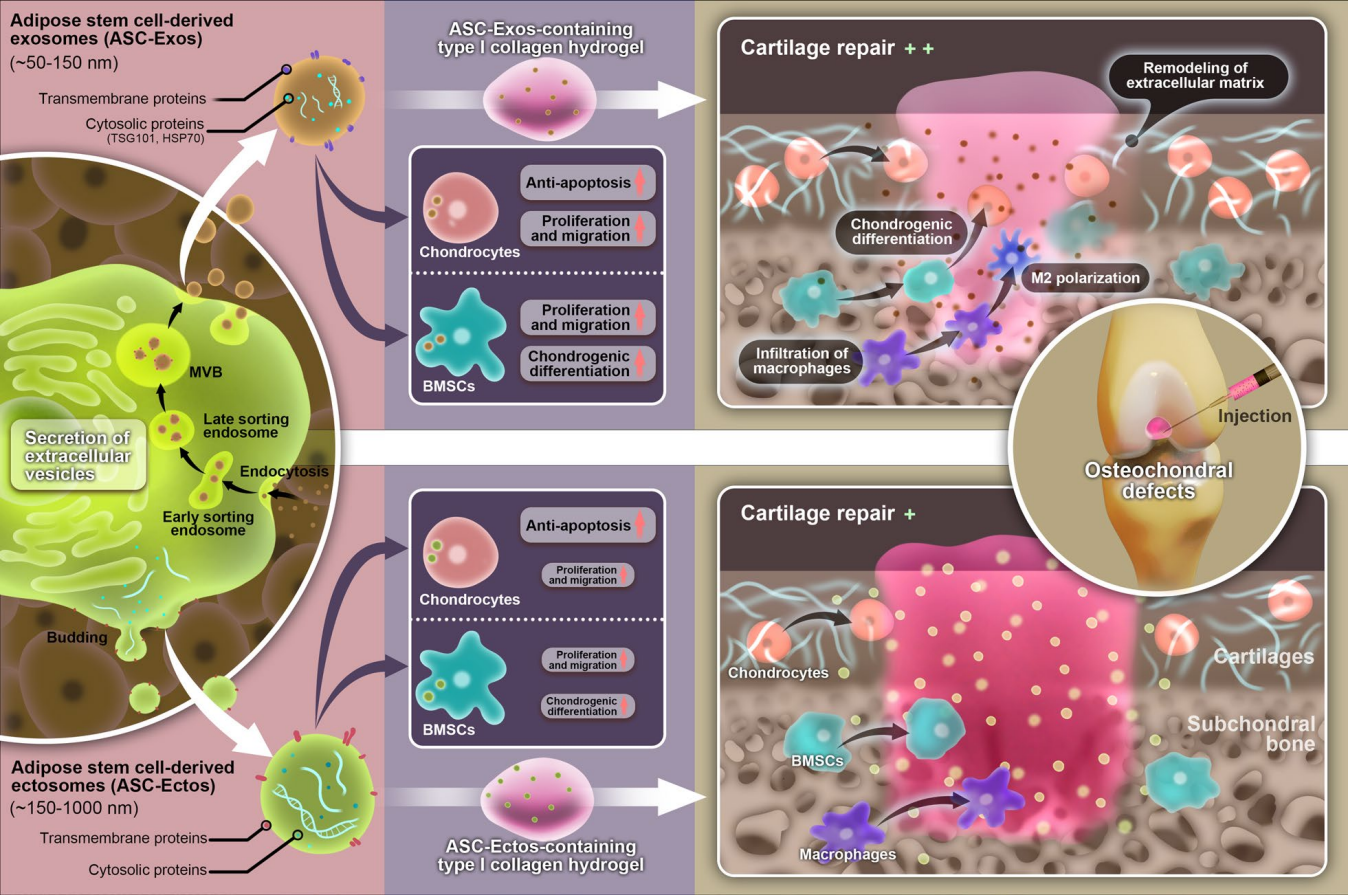
Schematic illustration of the experimental strategy and possible mechanism by which ASC-Exos promotes osteochondral regeneration more effectively than ASC-Ectos. The image was drawn by ourselves

In the present study, we compared for the first time the effect of exosomes and ectosomes derived from ASCs on cartilage regeneration. The cartilage repair effect of type I collagen hydrogel was amplified by the administration of both ASC-Exos and ASC-Ectos in a rat osteochondral model, but ASC-Exos had a greater therapeutic effect than ASC-Ectos. Furthermore, we demonstrated that the stronger repair effect of ASC-Exos may be attributed to its greater stimulation of migration and proliferation of chondrocytes and BMSCs, chondrogenesis of BMSCs, synthesis of ECM, and infiltration of M2 macrophages (Fig. 9).

EVs are composed of heterogeneous subgroups that are challenging to completely differentiate with current purification methods. Among these, although differential ultracentrifugation cannot fully separate ectosomes and exosomes, it is presently considered the gold standard method for EV purification [29, 30]. The NTA results indicated overlapping size distributions of the isolated ASC-Ectos and ASC-Exos, consistent with previous studies [19, 31]. This could account for negligible differences in many of the quantitative measures presented. Nevertheless, the vast majority of differences between the two EV populations led to the significant statistical variations in important measures. When separation techniques advance to allow the complete separation of the two EV subtypes, these results will need to be revalidated in the future.

It is well known that cartilage regeneration is an extremely complex process, involving the migration and proliferation of chondrocytes from adjacent cartilage as well as the migration, proliferation, and chondrogenesis of BMSCs from subchondral bone marrow [32]. In agreement with the literature, we observed that ASC-Exos promoted the proliferation and migration of chondrocytes and BMSCs in vitro [16, 17]. Furthermore, our study found that ASC-Ectos exhibited pro-migration and pro-proliferation effects on chondrocytes and BMSCs; however, the effect was weaker than that of ASC-Exos. The induction of chondrogenic differentiation of BMSCs in osteochondral defects is another important factor for accelerating the repair of cartilage and subchondral bone simultaneously [33]. Aligned with prior research findings, treatment with ASC-Exos of BMSCs resulted in enhanced chondrogenesis, including an increase in cell microsphere volume, collagen II, and aggrecan mRNA and protein expression in the ECM [34]. Additionally, the effects of ASC-Ecto on BMSCs were less pronounced than those of ASC-Exos.

Hydrogel scaffolds play a crucial role in cartilage regeneration by providing the required microenvironment and structure, compensating for the rapid clearance of certain drugs. The Gel group exhibited significantly superior reparative effects compared to the PBS group in macroscopic and histological assessments, aligning with the role of type I collagen hydrogels as a biocompatible scaffold for promoting cartilage defect repair [35, 36]. Enhanced performance in both aspects of assessment following integration with ASC-EVs suggests a promotive role of ASC-EVs in cartilage repair. This is consistent with the results reported in the literature, in which MSC-Exos laden in various hydrogel scaffolds promoted cartilage regeneration [15, 17, 37]. Furthermore, the structural integrity and quality of repaired cartilage in the Gel + Exo group were superior to those in the Gel + Ecto group, due partly to the stronger effect of ASC-Exos on chondrocytes and BMSCs in vitro compared to ASC-Ectos.

The evidence indicates that simultaneous regeneration of cartilage and subchondral bone is of paramount importance to the repair of osteochondral defects. Developing a single method to reconstruct both cartilage and subchondral bone within osteochondral defects remains a significant challenge due to physiological differences [38]. The micro-CT results showed that the subchondral bone in the Gel + Ecto and Gel + Exo groups had been well repaired. In addition, at 8 weeks, the Gel + Exo group had a higher BV/TV value and a lower Tb.Sp value than the Gel + Ecto group, indicating a better effect on bone regeneration. Curiously, there was no significant difference in quantitative results between the Gel and PBS groups, as collagen I hydrogels allow BMSCs to migrate, differentiate osteogenically, and have been utilized for bone repair in animal models [39, 40]. This is probably due to the fact that the microenvironment of pure bone tissue differs from that of subchondral bone, where there are dynamic interactions between cartilage and subchondral bone. The crosstalk between cartilage and subchondral bone may influence the differentiation of some endogenous MSCs into chondrocytes.

Dynamic remodeling of the ECM is essential for cartilage defects to heal and for cartilage homeostasis to be maintained [41]. A key difference between hyaline cartilage and fibrocartilage is the high content of aggrecan and type II collagen. Alcian blue staining shows long-chain sugar molecules, with larger blue-stained areas in Gel + Ecto and Gel + Exo groups compared to Gel and PBS groups [42]. Interestingly, at 8 weeks, aggrecan distribution and expression in Gel + Ecto and Gel + Exo groups were smaller and lower than at 4 weeks, respectively. This is because aggrecan is an early indicator of chondrogenic differentiation in the cartilage ECM [43]. Collagen fiber arrangement and accumulation were observed through polarized light and immunohistochemistry. Polarized light results showed improved arrangement of type II collagen fibers in the ASC-Exo group compared to ASC-Ecto, suggesting enhanced ECM maturity and biomechanics. The immunohistochemical images indicated that there was little difference between Gel + Ecto and Gel + Exo groups in the amount of type II collagen in the neotissue ECM. In spite of this, the uniform yellow–brown coloring of the Gel + Exo group indicates that type II collagen is distributed evenly, further supporting the hypothesis that the ECM of the Gel + Exo group is more mature than that of the Gel + Ecto group.

Injured joints are predisposed to continue arthritic degeneration, and for effective prevention of secondary degeneration, it is also necessary to improve the resistance of chondrocytes to excessive apoptosis in the local inflammatory environment [44]. TUNEL staining revealed increased apoptosis in repaired tissue compared to adjacent cartilage, potentially linked to local inflammation and catabolism. Gel + Ecto and Gel + Exo groups exhibited significantly fewer apoptotic cells than Gel alone in both regions, suggesting equal chondroprotective effects of ASC-Ectos and ASC-Exos. This contradicts previous reports indicating higher anti-apoptotic properties of MSC-Exos than MSC-Ectos at tested doses [14]. Discrepancies may arise from variations in EV isolation protocols, MSC sources, culture conditions, apoptotic induction agents, and therapeutic EV doses. Macrophage infiltration plays a crucial role in cartilage regeneration, influencing homeostasis and regulating inflammatory and regenerative processes [45]. Specifically, M2 macrophages, characterized by the specific expression of CD206 and Arginase-1, are reported to mitigate the inflammatory response and promote regeneration [46, 47]. In our study, we found that ASC-Exos induced higher infiltration of regenerative M2 macrophages at the defect site with a concomitant increase in the anti-inflammatory marker Arginase-1. This aligns with reported MSC-Exos properties promoting M2 macrophage infiltration in cartilage repair [48]. However, ASC-Ectos did not appear to affect the induction of M2 macrophages during cartilage repair, as was observed in our previous study conducted under tendinopathy conditions.

As a key component of ASC-EVs, protein plays a critical functional role. Previous studies have demonstrated that different proteins are enriched in ectosomes and exosomes, as reflected in the cluster analysis [19, 49]. Among the most up-regulated proteins, those like Lama4 and Nid2 implicated in chondrogenesis, and Lgals3bp associated with osteogenesis, may contribute to cartilage regeneration [50, 51]. Furthermore, Ttyh3 positively correlates with cell proliferation, migration, and immune cell function [52, 53]. In mice with collagen-induced arthritis, PTX3 administration significantly reduced cartilage and bone destruction [54]. The enrichment analysis results suggested that enriched GO terms, such as cell migration, protein binding, and cell surface, as well as enriched KEGG terms, including endocytosis, necroptosis, and the MAPK signaling pathway, are involved in regulating the synthesis and degradation of the ECM, promoting chondrogenic differentiation and proliferation, and playing an essential role in cartilage repair [55–58]. The PPI network results revealed interactions among target proteins, providing further insight into the key proteins involved in the superior cartilage repair effects of ASC-Exos compared to ASC-Ectos in the future.

The present study has some limitations that should be acknowledged. First, the release kinetics of the two EVs from the hydrogel were unclear, which may contribute to the differences in cartilage repair efficacy. Second, the exact in vivo mechanism by which ASC-EVs promote cartilage repair cannot be definitively confirmed at present due to the lack of in vivo tracking of endogenous BMSCs and chondrocytes, as well as the chondrogenic differentiation of BMSCs. Third, the in vivo follow-up time in this study is relatively short compared to the lengthy process of restoring and modeling the structure of the osteochondral defect. Fourth, a "whole EV" control group needs to be included in further studies to investigate whether the combined use of Exos and Ectos exhibits potential synergistic effects, which is crucial for understanding their future therapeutic applications. Finally, there may be inter-species differences between EVs from human and animal cell sources, which can influence experimental results. Therefore, it is necessary to revalidate the conclusions in humans before clinical application.

## Conclusions

In conclusion, as demonstrated in in vitro studies, both subtypes of EVs could facilitate the viability and migration of chondrocytes and BMSCs, suppress chondrocyte apoptosis, and promote chondrogenic differentiation of BMSCs. Specifically, ASC-Exos showed higher bioactivity than ASC-Ectos at the same concentration, except for their anti-apoptosis effects. Furthermore, in vivo studies demonstrated that both EVs possessed favorable capacities for cartilage regeneration in an experimental rat model of osteochondral defect, although ASC-Exos showed superior performance. Histological analysis revealed that the underlying mechanisms for the enhanced bioactivity of ASC-Exos could be attributed to the enhanced cartilage ECM synthesis, accelerated subchondral bone reconstruction, inhibited chondrocyte apoptosis, and increased M2 macrophage infiltration. Hence, our study indicates that ASC-Exos containing type I collagen hydrogels are a promising strategy for cartilage regeneration and provide new insights and guidance for the clinical transformation of MSC-EVs in the treatment of cartilage injury.

### Supplementary Information

**Additional file 1.** **Fig. S1.** Isolation and characterization of ASC-Exos and ASC-Ectos. (A) Process flow diagram for the isolation and purification of ASC-Exos and ASC-Ectos. (B) Visualization of the morphology of ASC-Exos and ASC-Ectos using transmission electron microscopy. (C) NTA measurements of the particle size distribution of ASC-Exos and ASC-Ectos.

**Additional file 2.** **Fig. S2.** Top 9 most up-regulated proteins.

**Additional file 3. Fig. S3**. Top 9 most down-regulated proteins.

**Additional file 4.** **Fig. S4**. GO analysis. BP, biological processes; CC, cellular components; MF, molecular functions.

**Additional file 5.** **Fig. S5**. KEGG pathways enrichment analysis.

**Additional file 6.** **Fig. S6.** Protein-protein interactions (PPI) of the targets were analyzed using the PPI network. (a) Target protein direct interaction network. (b) The target protein interacts with all other identified proteins. Blue nodes indicate up-regulated proteins with the most intense expression; cyan nodes indicate down-regulated proteins with the most intense expression; gray nodes are the other proteins identified. The node size is expressed as the value of degree.

**Additional file 7. Table S1**. International Cartilage Repair Society (ICRS) score for macroscopic assessment for cartilage repair.

**Additional file 8**. **Table S2**. The modified O’Driscoll histologic (MODS) score for histologic assessment for cartilage repair.

**Additional file 9.** **Table S3**. The ICRS scores and MODS scores of each group.

## Data Availability

The mass spectrometry proteomics data have been deposited to the ProteomeXchange Consortium (http://proteomecentral.proteomexchange.org) via the iProX partner repository with the dataset identifier PXD041283.

## Abbreviations

EVs: Extracellular vesicles
MSCs: Mesenchymal stem cells
BMSCs: Bone marrow-derived MSCs
ASCs: Adipose-derived stem cells
ASC-Exos: ASC-derived exosomes
ASC-Ectos: ASC-derived ectosomes
BV/TV: Bone volume to tissue volume
BMD: Bone mineral density
Tb.Sp: Trabecular separation
NSAIDs: Non-steroidal anti-inflammatory drugs
HA: Hyaluronic acid
ACI: Autologous chondrocyte implantation
MACI: Matrix-assisted autologous chondrocyte implantation
MSC-EVs: MSC-derived EVs
MVBs: Multivesicular bodies
MSC-Exos: MSC-derived exosomes
MSC-Ectos: MSC-derived ectosomes
NTA: Nanoparticle tracking analysis
TEM: Transmission electron microscope
FBS: Fetal bovine serum
PBS: Phosphate-buffered saline
PFA: Paraformaldehyde
DAPI: 4′,6-Diamidino-2-phenylindol
CCK8: Cell counting kit-8
APC: Allophycocyanin
PI: Propidium iodide
qRT-PCR: Quantitative RT polymerase chain reaction
WB: Western blotting
RIPA: Radioimmunoprecipitation
BCA: Bicinchoninic acid
SDS-PAGE: Sodium dodecyl sulfate–polyacrylamide gel electrophoresis
PVDF: Polyvinylidene fluoride
ECL: Enhanced chemiluminescence
Gel: Hydrogel
Gel + Ectos: Hydrogel containing ectosomes
Gel + Exos: Hydrogel containing exosomes
ICRS: International Cartilage Repair Society
Micro-CT: Micro-computed tomography
EDTA: Ethylenediaminetetraacetic acid
HE: Hematoxylin and eosin
DAB: Diaminobenzidine
TUNEL: Terminal deoxynucleotidyl transferase dUTP nick-end labeling
ANOVA: One-way analysis of variance
SD: Standard error
SMSCs: Synovium-derived MSCs
ECM: Extracellular matrix
