# Supplementary material for: Comparison of the ability of exosomes and ectosomes derived from adipose-derived stromal cells to promote cartilage regeneration in a rat osteochondral defect model
Source: Stem Cell Res Ther. 2024 Jan 17;15:18. doi: 10.1186/s13287-024-03632-4 (PMC10792834; doi:10.1186/s13287-024-03632-4)
Supplement: Supplementary file 1 — Additional file 1. Fig. S1. Isolation and characterization of ASC-Exos and ASC-Ectos. (A) Process flow diagram for the isolation and purification of ASC-Exos and ASC-Ectos. (B) Visualization of the morphology of ASC-Exos and ASC-Ectos using transmission electron microscopy. (C) NTA measurements of the particle size distribution of ASC-Exos and ASC-Ectos. [file 13287_2024_3632_MOESM1_ESM.docx]

**
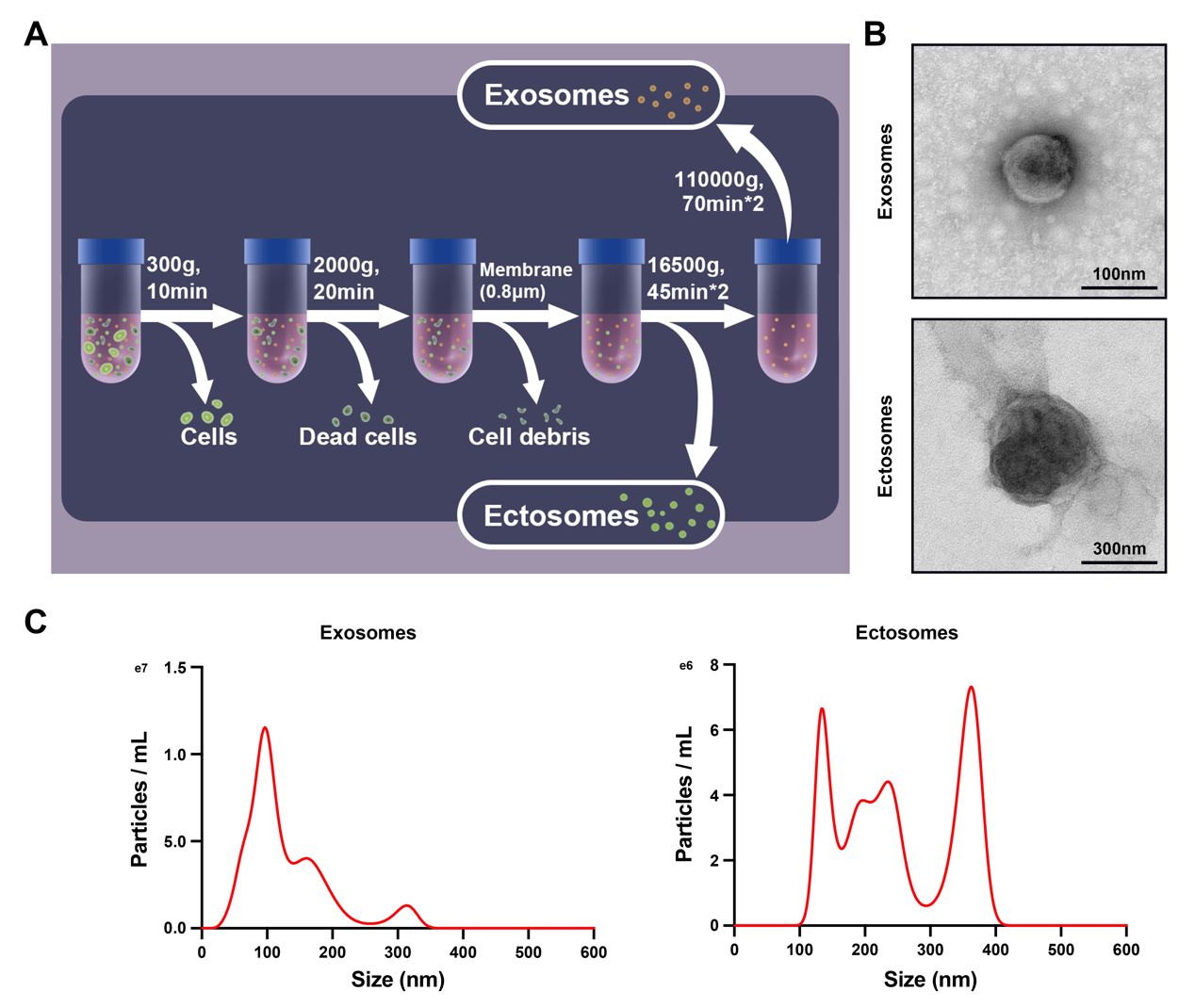
**

**Supplementary Fig. 1.** Isolation and characterization of ASC-Exos and ASC-Ectos. (A) Process flow diagram for the isolation and purification of ASC-Exos and ASC-Ectos. (B) Visualization of the morphology of ASC-Exos and ASC-Ectos using transmission electron microscopy. (C) NTA measurements of the particle size distribution of ASC-Exos and ASC-Ectos.
