# Supplementary figures and images for: Comparison of the ability of exosomes and ectosomes derived from adipose-derived stromal cells to promote cartilage regeneration in a rat osteochondral defect model
Source: Stem Cell Res Ther. 2024 Jan 17;15:18. doi: 10.1186/s13287-024-03632-4 (PMC10792834; doi:10.1186/s13287-024-03632-4)

**
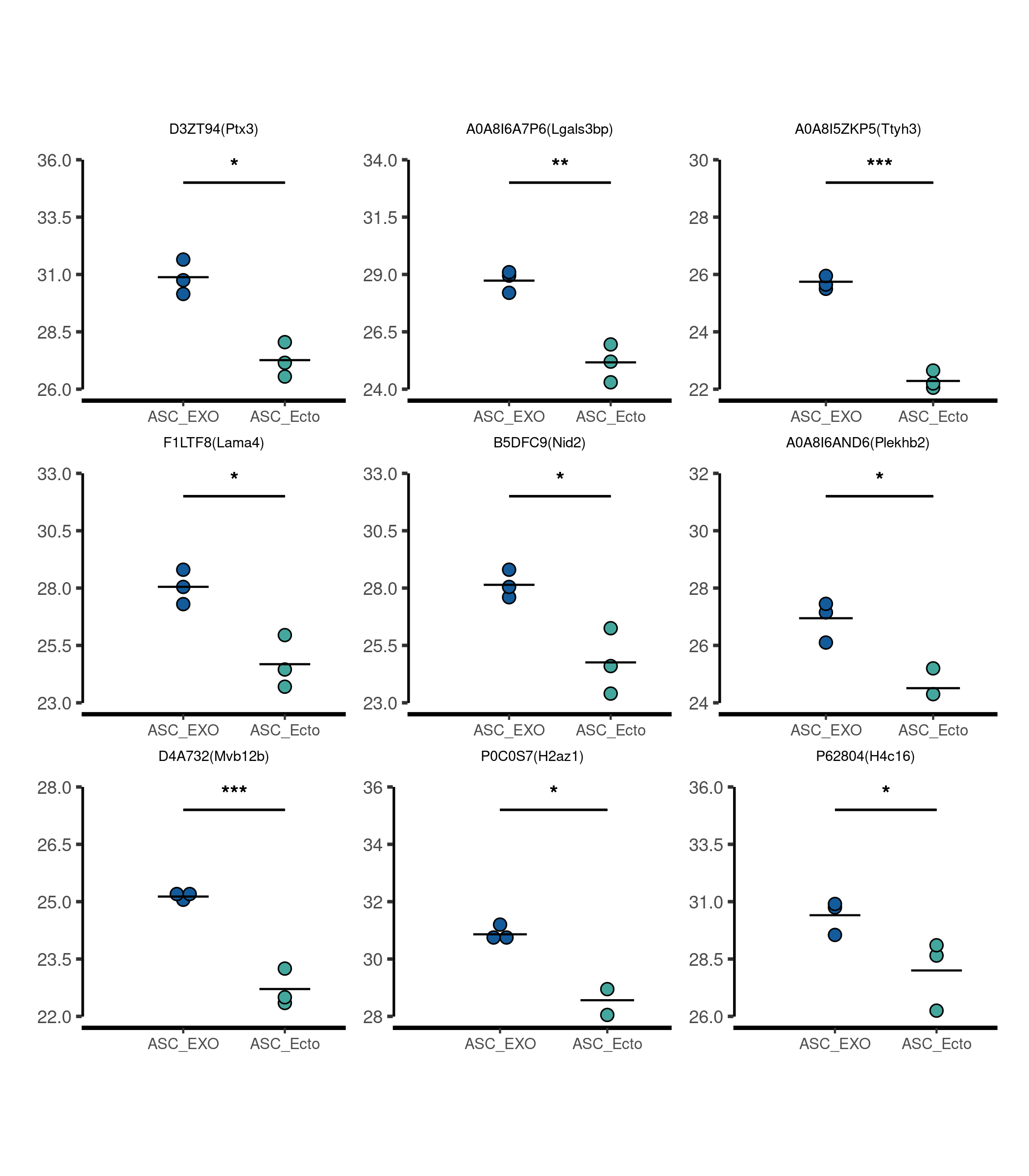
**

**Supplementary Fig. 2.** Top 9 most up-regulated proteins.

Supplement: Supplementary file 2 — Additional file 2. Fig. S2. Top 9 most up-regulated proteins. [file 13287_2024_3632_MOESM2_ESM.docx]

**
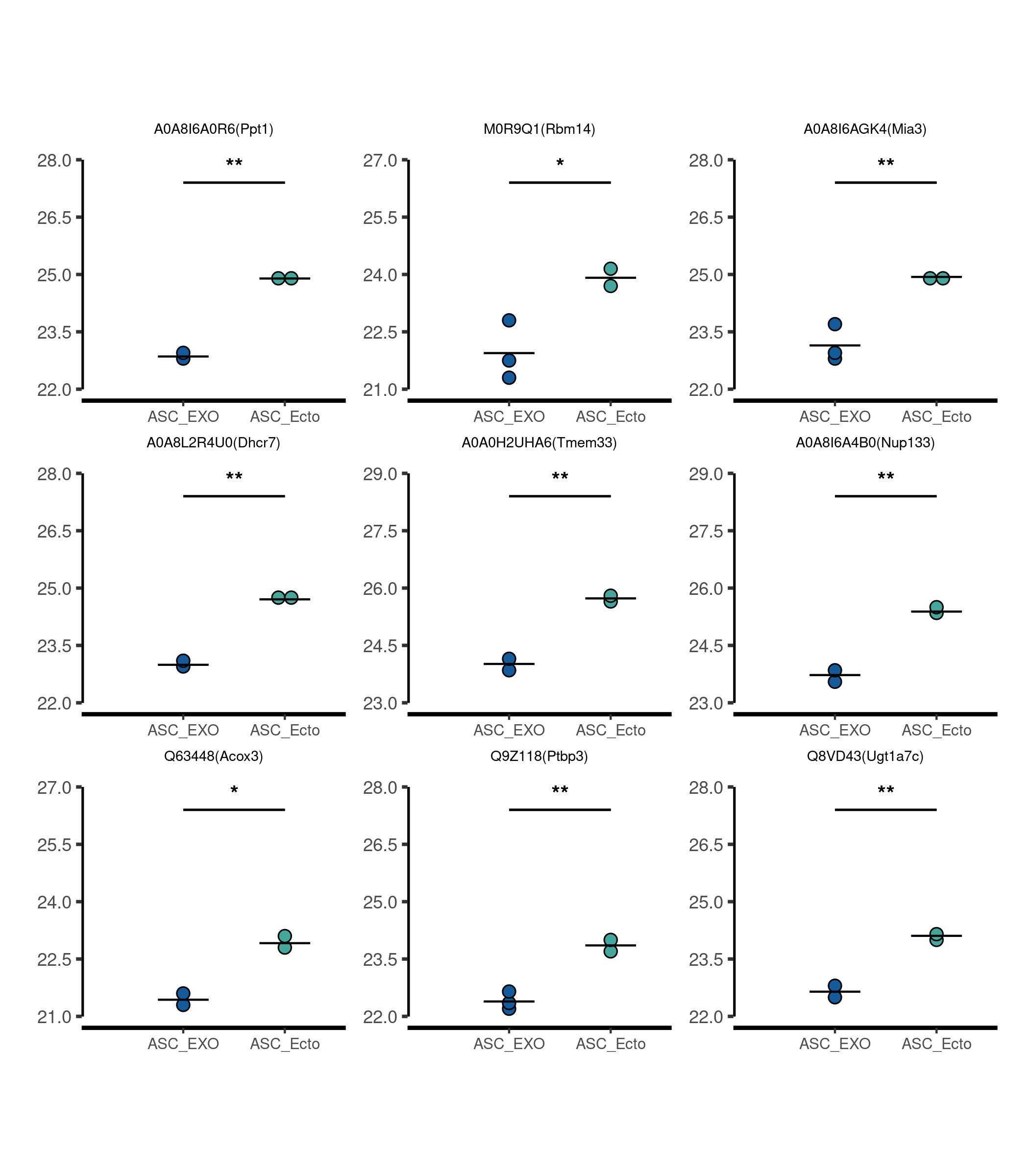
**

**Supplementary Fig. 3.** Top 9 most down-regulated proteins.

Supplement: Supplementary file 3 — Additional file 3. Fig. S3. Top 9 most down-regulated proteins. [file 13287_2024_3632_MOESM3_ESM.docx]

**
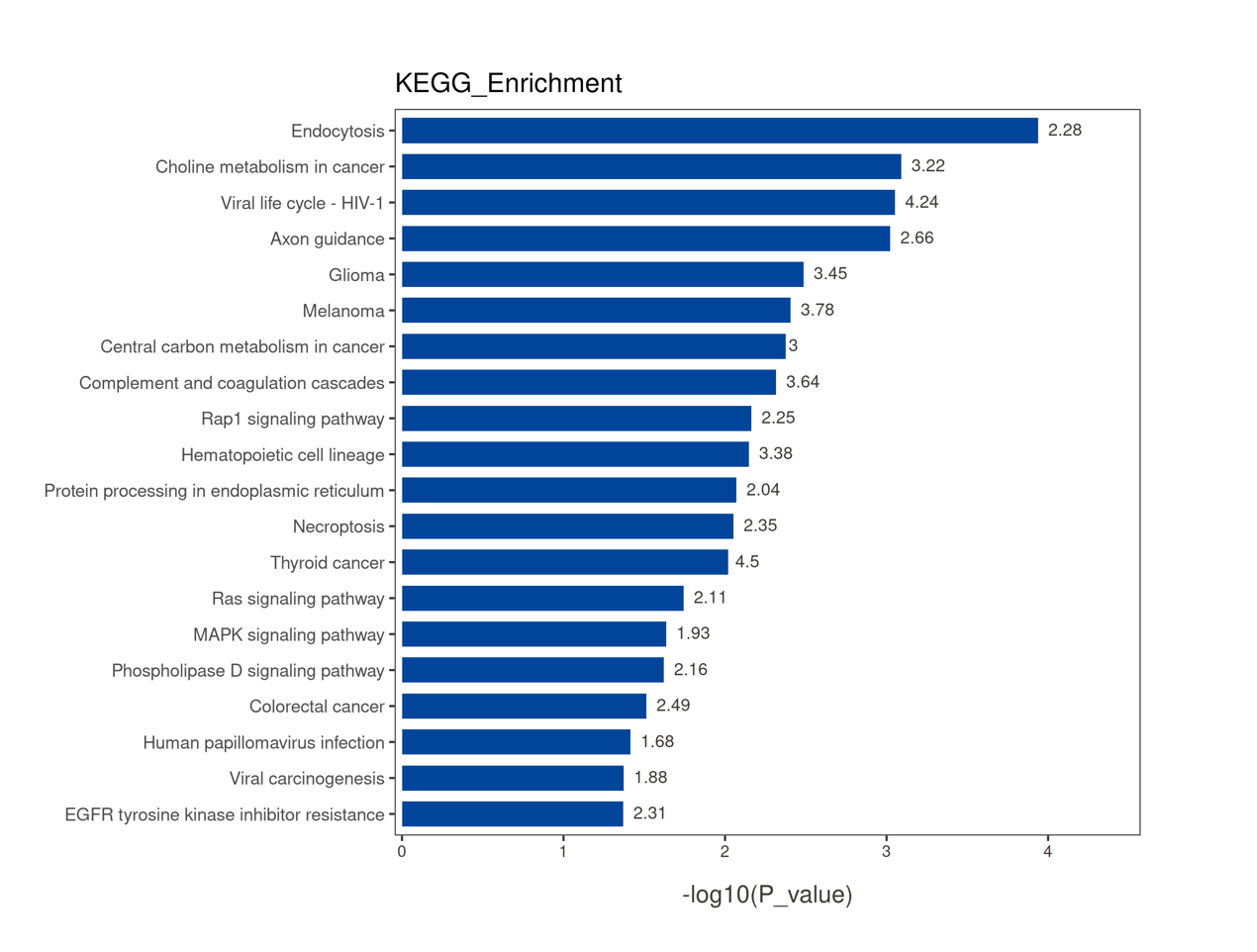
**

**Supplementary Fig. 5.** KEGG pathways enrichment analysis.

Supplement: Supplementary file 5 — Additional file 5. Fig. S5. KEGG pathways enrichment analysis. [file 13287_2024_3632_MOESM5_ESM.docx]
