# Supplementary material for: Comparison of the ability of exosomes and ectosomes derived from adipose-derived stromal cells to promote cartilage regeneration in a rat osteochondral defect model
Source: Stem Cell Res Ther. 2024 Jan 17;15:18. doi: 10.1186/s13287-024-03632-4 (PMC10792834; doi:10.1186/s13287-024-03632-4)
Supplement: Supplementary file 4 — Additional file 4. Fig. S4. GO analysis. BP, biological processes; CC, cellular components; MF, molecular functions. [file 13287_2024_3632_MOESM4_ESM.docx]

**
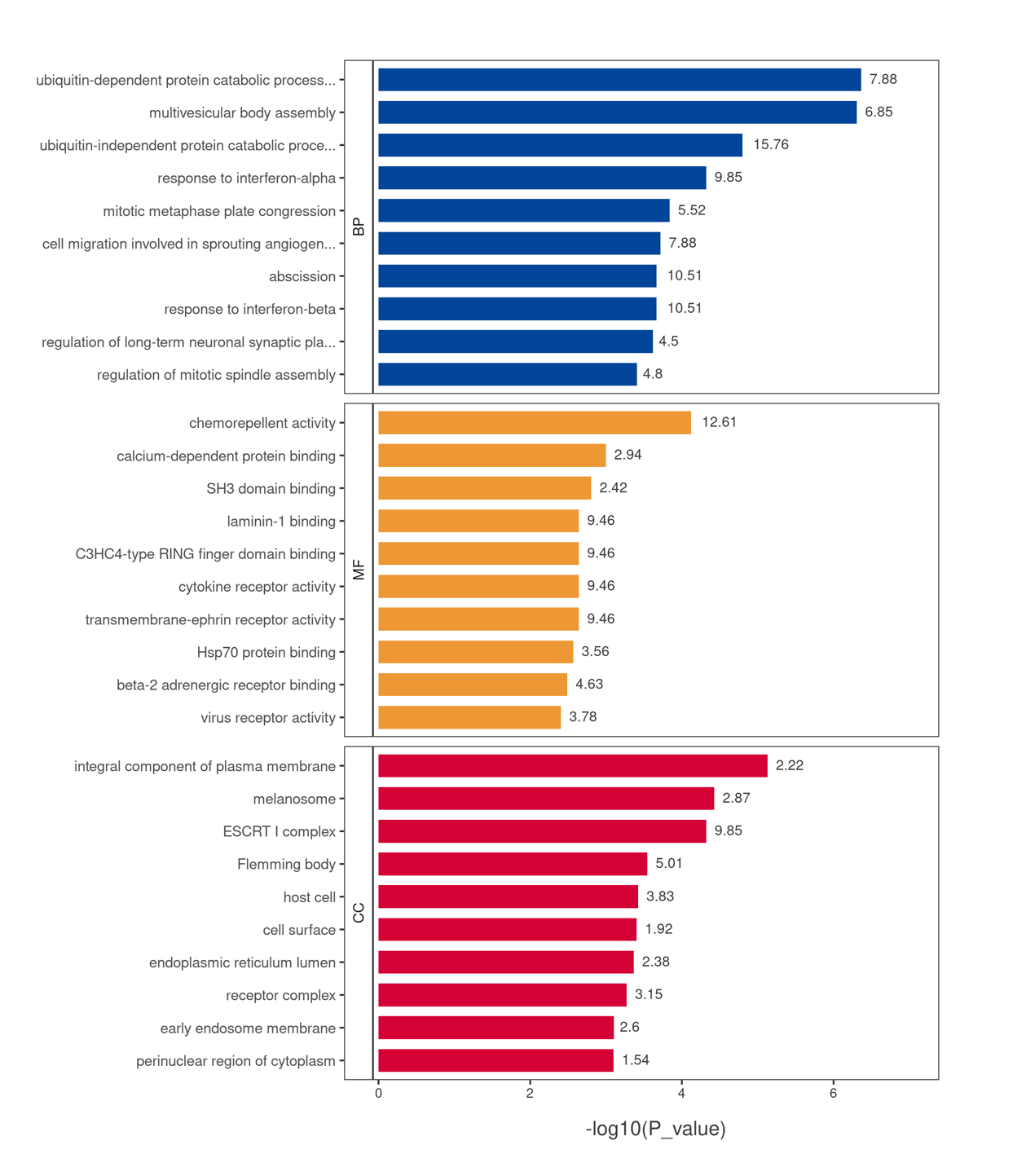
**

**Supplementary Fig. 4.** GO analysis. BP, biological processes; CC, cellular components; MF, molecular functions.
