# Supplementary material for: Comparison of the ability of exosomes and ectosomes derived from adipose-derived stromal cells to promote cartilage regeneration in a rat osteochondral defect model
Source: Stem Cell Res Ther. 2024 Jan 17;15:18. doi: 10.1186/s13287-024-03632-4 (PMC10792834; doi:10.1186/s13287-024-03632-4)
Supplement: Supplementary file 6 — Additional file 6. Fig. S6. Protein-protein interactions (PPI) of the targets were analyzed using the PPI network. (a) Target protein direct interaction network. (b) The target protein interacts with all other identified proteins. Blue nodes indicate up-regulated proteins with the most intense expression; cyan nodes indicate down-regulated proteins with the most intense expression; gray nodes are the other proteins identified. The node size is expressed as the value of degree. [file 13287_2024_3632_MOESM6_ESM.docx]

**
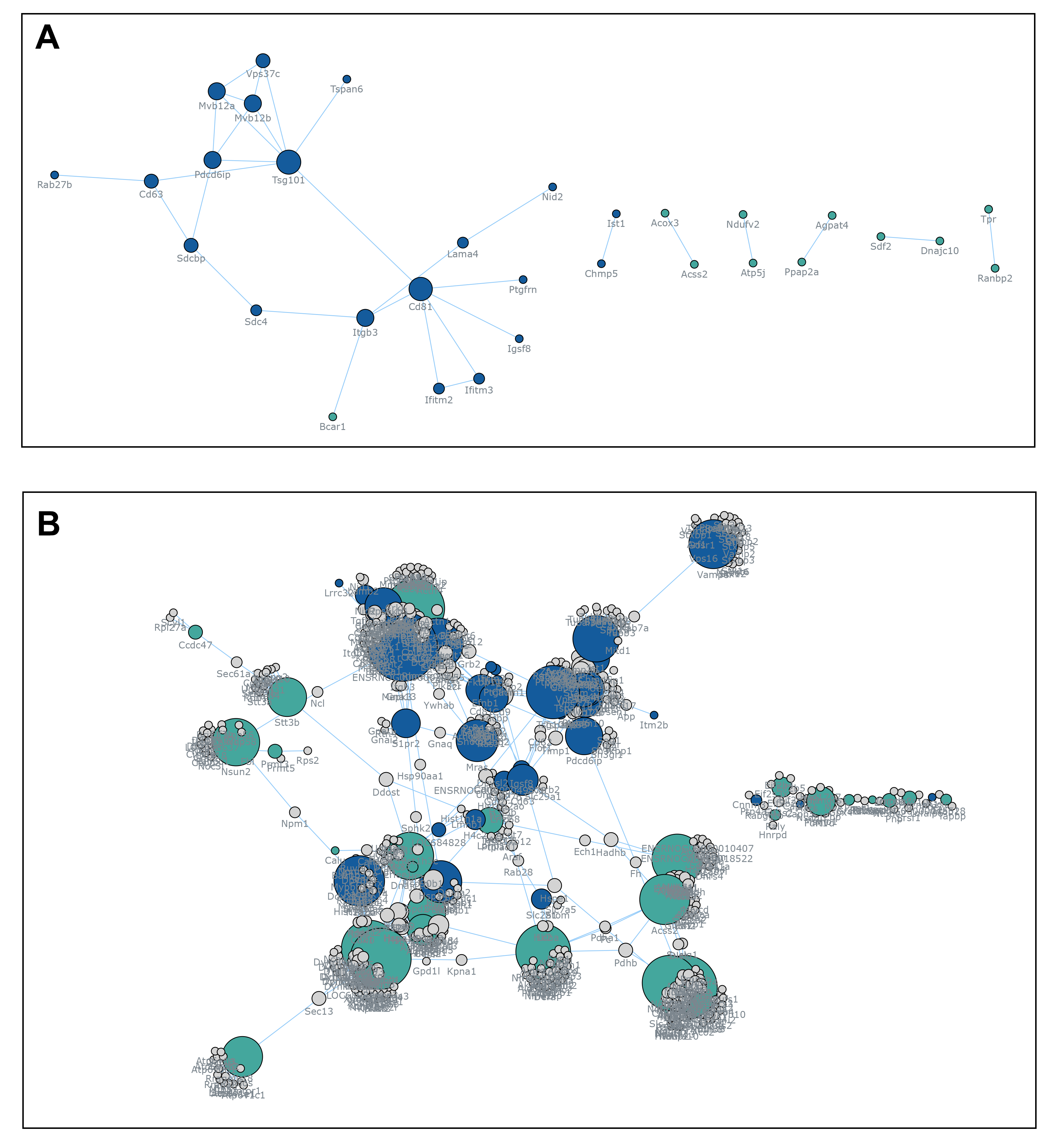
**

**Supplementary Fig. 6.** Protein-protein interactions (PPI) of the targets were analyzed using the PPI network. (**a**) Direct interaction network between target proteins. (**b**) The target protein interacts with all other identified proteins. Blue nodes indicate up-regulated proteins with the most intense expression; cyan nodes indicate down-regulated proteins with the most intense expression; gray nodes are the other proteins identified. The node size is expressed as the value of degree.
