# Supplementary material for: Comparison of the ability of exosomes and ectosomes derived from adipose-derived stromal cells to promote cartilage regeneration in a rat osteochondral defect model
Source: Stem Cell Res Ther. 2024 Jan 17;15:18. doi: 10.1186/s13287-024-03632-4 (PMC10792834; doi:10.1186/s13287-024-03632-4)
Supplement: Supplementary file 7 — Additional file 7. Table S1. International Cartilage Repair Society (ICRS) score for macroscopic assessment for cartilage repair. [file 13287_2024_3632_MOESM7_ESM.docx]

**Supplementary Table 1.** International Cartilage Repair Society (ICRS) score for macroscopic assessment for cartilage repair.

| Criteria | | Points |
| --- | --- | --- |
| I. Degree of defect repair | |  |
|  | Level with surrounding cartilage | 4 |
|  | 75% repair of defect depth | 3 |
|  | 50% repair of defect depth | 2 |
|  | 25% repair of defect depth | 1 |
|  | 0% repair of defect depth | 0 |
| II. Integration to border zone | |  |
|  | Complete integration with surrounding cartilage | 4 |
|  | Demarcating border <1 mm | 3 |
|  | 3/4 of graft integrated, 1/4 with a notable border >1 mm width | 2 |
|  | 1/2 of graft integrated with surrounding cartilage,  1/2 with a notable border >1 mm | 1 |
|  | From no contact to 1/4 of graft integrated with surrounding cartilage | 0 |
| III. Macroscopic appearance | |  |
|  | Intact smooth surface | 4 |
|  | Fibrillated surface | 3 |
|  | Small, scattered fissures or cracks | 2 |
|  | Several, small or few but large fissures | 1 |
|  | Total degeneration of grafted area | 0 |
| IV. Overall score | |  |
|  | Grade I: normal | 12 |
|  | Grade II: nearly normal | 11-8 |
|  | Grade III: abnormal | 7-4 |
|  | Grade IV: severely abnormal | 3-1 |
