# Supplementary material for: Comparison of the ability of exosomes and ectosomes derived from adipose-derived stromal cells to promote cartilage regeneration in a rat osteochondral defect model
Source: Stem Cell Res Ther. 2024 Jan 17;15:18. doi: 10.1186/s13287-024-03632-4 (PMC10792834; doi:10.1186/s13287-024-03632-4)
Supplement: Supplementary file 8 — Additional file 8. Table S2. The modified O’Driscoll histologic (MODS) score for histologic assessment for cartilage repair. [file 13287_2024_3632_MOESM8_ESM.docx]

**Supplementary Table 2**. The modified O’Driscoll histologic (MODS) score for histologic assessment for cartilage repair.

| Characteristic |  | Score |
| --- | --- | --- |
| I. Hyaline cartilage (%) | |  |
|  | 80–100 | 8 |
|  | 60–80 | 6 |
|  | 40–60 | 4 |
|  | 20–40 | 2 |
|  | 0–20 | 0 |
| II. Structural characteristics | |  |
| A. Surface irregularity | |  |
|  | Smooth and intact | 2 |
|  | Fissures | 1 |
|  | Severe disruption, fibrillation | 0 |
| B. Structural integrity | |  |
|  | Normal | 2 |
|  | Slight disruption, including cysts | 1 |
|  | Severe lack of integration | 0 |
| C. Thickness | |  |
|  | 100% of normal adjacent cartilage | 2 |
|  | 50% to 100% or thicker than normal | 1 |
|  | 0–50% | 0 |
| D. Bonding to adjacent cartilage | |  |
|  | Bonded at both ends of graft | 2 |
|  | Bonded at one end/partially both ends | 1 |
|  | Not bonded | 0 |
| III. Freedom from cellular changes of degeneration | |  |
|  | Normal cellularity, no clusters | 2 |
|  | Slight hypocellularity, <25% chondrocyte clusters | 1 |
|  | Moderate hypocellularity, >25% clusters | 0 |
| IV. Freedom from degenerate changes in adjacent cartilage | |  |
|  | Normal cellularity, no clusters, normal staining | 3 |
|  | Normal cellularity, mild clusters, moderate staining | 2 |
|  | Mild or mod hypocellularity, slight staining | 1 |
|  | Severe hypocellularity, slight staining | 0 |
| V. Reconstitution of subchondral bone | |  |
|  | Complete reconstitution | 2 |
|  | Greater than 50% recon | 1 |
|  | 50% or less recon | 0 |
| VI. Bonding of repair cartilage to de novo subchondral bone | |  |
|  | Complete and uninterrupted | 2 |
|  | <100% but >50% recon | 1 |
|  | <50% complete | 0 |
| VII. Safranin O staining | |  |
|  | >80% homogeneous positive stain | 2 |
|  | 40%–80% homogeneous positive stain | 1 |
|  | <40% homogeneous positive stain | 0 |
| Total score |  | Max27 |
