# Supplementary material for: Comparison of the ability of exosomes and ectosomes derived from adipose-derived stromal cells to promote cartilage regeneration in a rat osteochondral defect model
Source: Stem Cell Res Ther. 2024 Jan 17;15:18. doi: 10.1186/s13287-024-03632-4 (PMC10792834; doi:10.1186/s13287-024-03632-4)
Supplement: Supplementary file 9 — Additional file 9. Table S3. The ICRS scores and MODS scores of each group. [file 13287_2024_3632_MOESM9_ESM.docx]

**Supplementary Table 3**. The ICRS scores and MODS scores of each group.

| Group  Time | PBS | Gel | Gel+Ectos | Gel+Exos |
| --- | --- | --- | --- | --- |
| ICRS scores | | | | |
| 4 weeks | 5.72±0.913 | 7.33±0.665 | 8.38±0.780 | 9.38±0.891 |
| 8 weeks | 6.00±1.040 | 8.33±0.693 | 9.57±0.498 | 11.0±0.470 |
| MODS scores | | | | |
| 4 weeks | 11.8±1.70 | 14.0±1.01 | 15.7±1.60 | 17.3±1.39 |
| 8 weeks | 13.4±1.37 | 16.3±0.859 | 19.8±2.11 | 23.5±2.58 |
